# Transcriptional transactivation turns human iPSC-derived macrophages into an adenovirus-producing cell state

**DOI:** 10.1128/jvi.00392-26

**Published:** 2026-05-04

**Authors:** Maarit Suomalainen, Walther Haenseler, Jonas Kolibius, Andreas Plückthun, Patrick Hearing, Urs F. Greber

**Affiliations:** 1 Department of Molecular Life Sciences, University of Zurich 400018, Zurich, Switzerland; 2 Department of Biology, Institute of Molecular Systems Biology, ETH Zurich 30845 , Zurich, Switzerland; 3 URPP Adaptive Brain Circuits in Development and Learning, University of Zurich 27217, Zurich, Switzerland; 4 Department of Biochemistry, University of Zurich 27217, Zurich, Switzerland; 5 Department of Microbiology and Immunology, School of Medicine, Stony Brook University 12301, Stony Brook, New York, USA; Tufts University School of Medicine, Boston, Massachusetts, USA

**Keywords:** cell state, macrophage, single cell infection biology, viral entry and transcription, viral persistence and transactivation

## Abstract

Macrophages conduct first-in-line defense against pathogens, including human adenovirus (AdV). AdVs cause respiratory disease, persist in immune cells, and, upon reactivation, are life-threatening to immunocompromised individuals. Here, our single-cell, single-virus experiments showed that AdV-type-C5 entry into human induced-pluripotent stem cell-derived macrophages is attenuated at cell binding and endosomal escape. A significant fraction (~30%) of the double-stranded viral DNA (vDNA) reaches the cell nucleus; however, it failed to efficiently express the immediate-early viral epigenetic regulator E1A. E1A transcription of silenced vDNA was rescued by E1A expression from a heterologous promoter of a superinfecting AdV, and allowed for full viral replication and progeny formation, even days post-infection, indicating long-lived infectivity of dormant vDNA. Bulk RNA-seq analyses showed that attenuated single AdV-C5 infections upregulated signaling, defense, and proinflammatory genes, whereas productive coinfections upregulated DNA replication and signaling pathways. Together, our data demonstrate that macrophages are a Trojan horse for AdV, notably independent of interferon, raising the possibility that macrophages function as a reservoir for AdV *in vivo* and reactivate dormant virus via epigenetic signals.

**IMPORTANCE:**

AdV are widespread, cause severe respiratory disease, persist in immune cells, and, upon reactivation, cause life-threatening conditions in immunocompromised individuals. Here, we show that human macrophages are either protected or susceptible to AdV, depending on the cell state, notably in an interferon-independent manner. The decisive cell state switch is the viral immediate-early transcription modulator E1A, which turns a repressive state into a permissive one and allows for the transactivation of dormant AdV-C5 genomes and viral progeny production. The data raise the possibility that macrophages are a hub for AdV persistence and epigenetic reactivation *in vivo*, in line with the notion that these cells resist immune clearance and serve as reservoirs for HIV, herpesvirus, SARS-CoV-2, or rubella virus infections.

## INTRODUCTION

Differentiated macrophages act in the first line of defense against viruses, contribute to excessive tissue injury, or enhance infection of nearby cells (1, 2). They are important for immune control but also resist immune clearance, supporting their role as a reservoir for HIV retroviruses, RNA viruses including SARS-CoV-2 or rubella, and double-stranded DNA viruses, such as herpesviruses (3–7).

In immunoproficient individuals, larger DNA viruses cause acute and persistent infections, often controlled by temporal viral gene expression programs in the cell nucleus, as in the case of adenoviruses (AdV) and herpesviruses (8, 9), but also of small DNA tumor viruses of the polyoma and papilloma virus families (10, 11). Seven human AdV species have been described (A–G), and six genotypes of species C, AdV-C1, C2, C5, C6, C57, and C89 (12). They clinically account for ~15% of upper respiratory tract infections and ~5% of lower respiratory tract infections (13). Epidemiologic and clinical data show that upon viremia, AdV-C5 causes persistent infections, which can reactivate, affect the central nervous system, and become life-threatening to immunosuppressed children and adults (14, 15).

AdV particles carry a double-stranded DNA genome (vDNA) of ~34–36 kbp enclosed in a non-enveloped icosahedral capsid (16). They have evolved a temporally orchestrated gene expression program, with immediate-early, early, and late transcription units (8). The immediate-early AdV promoter gives rise to alternatively spliced 13S and 12S E1A transcripts encoding the transcriptional regulators large and small E1A of 289 and 243 amino acids, respectively, both lacking DNA-binding activity (17). Large and small E1A epigenetically activate all the AdV promoters and a range of cellular promoters and rewire the cell cycle state and the metabolome (e.g., 18–22). E1A acts through recruiting histone acetyltransferases and chromatin-remodeling complexes, such as p300/CBP, increasing chromatin accessibility for transcription factors (19, 23, 24). In tissue culture cell lines, E1A transcription commences after delivery of the vDNA into the nucleus and rapidly yields high transcript numbers (25, 26). In primary human bronchial epithelial cells or TERT-immortalized normal human diploid fibroblasts (HDF-TERT), interferon (IFN) represses E1A transcription by recruiting the retinoblastoma protein (Rb)/E2F/histone deacetylase repressor complexes to the E1A enhancer/promoter (e/p) (27, 28), while a viral feedforward loop through the unfolded protein response sensor Ire1a maintains at least low levels of E1A expression and AdV-C5 persistence (29). The importance of epigenetic E1A regulation is underscored by the notion that a preexisting cell state with a memory effect of about 9 weeks co-determines whether the E1A-e/p has high or low activity (30).

Although the expression levels of E1A are key to lytic, persistent, latent, or abortive infections, additional processes independent of E1A also affect the infection outcome. AdV infection in tissue culture epithelial cell lines, such as A549, is lytic and efficient, with high progeny yield, whereas, for example, B and T cells generally exhibit low susceptibility to AdVs (31, 32), but gastrointestinal tract lymphocytes are important reservoir cells for persistent AdV infections (14).

It is less known if other hematopoietic cells, such as macrophages, harbor persistently active AdV genomes. Macrophages replicate AdV at low levels (33), and their interactions with AdV trigger an instant release of proinflammatory cytokines and chemokines *in vivo* and *in vitro* (3, 34–36), including the chemotactic cytokine CXCL8 (interleukin 8), a neutrophil activator released from monocytes (37). A CXCL8 signaling cascade relocates AdV receptors CAR and ανβ3 integrins to the apical plasma membrane of polarized epithelial cells, enhancing virus entry and progeny production (35, 38). A recent humanized mouse model harboring human hematopoietic stem and progenitor cells (HSPCs) showed that AdV-C2 establishes an asymptomatic persistent infection in bone marrow and peripheral blood cells with low levels of E1A expression (39). Bone marrow harbors high amounts of resident macrophages, osteomacs, with a key role in supporting HSPCs. HSPCs give rise to B and T cells, which can harbor persisting AdV-C in the human gastrointestinal mucosa or *in vitro* (32, 40).

Here, we mechanistically analyze AdV persistence in macrophages *in vitro* and demonstrate that human induced-pluripotent stem cell (hiPSC)-derived macrophages (41–43) can be persistently infected with AdV-C5 and reactivated by the expression of the viral epigenetic regulator E1A. The gene expression profile indicated that their susceptibility state is epigenetically controlled, notably in an interferon-independent manner. These observations raise the possibility that macrophages, including monocytes and tissue-resident macrophages, such as osteomacs, are a hub for AdV persistence and epigenetic reactivation *in vivo*, resonating the notion that macrophages have been shown to resist immune clearance in case of HIV, herpesvirus, SARS-CoV-2, or rubella virus infections.

## RESULTS

### Inefficient AdV-C5 transduction of hiPSC-derived macrophages

**Fig 1:**
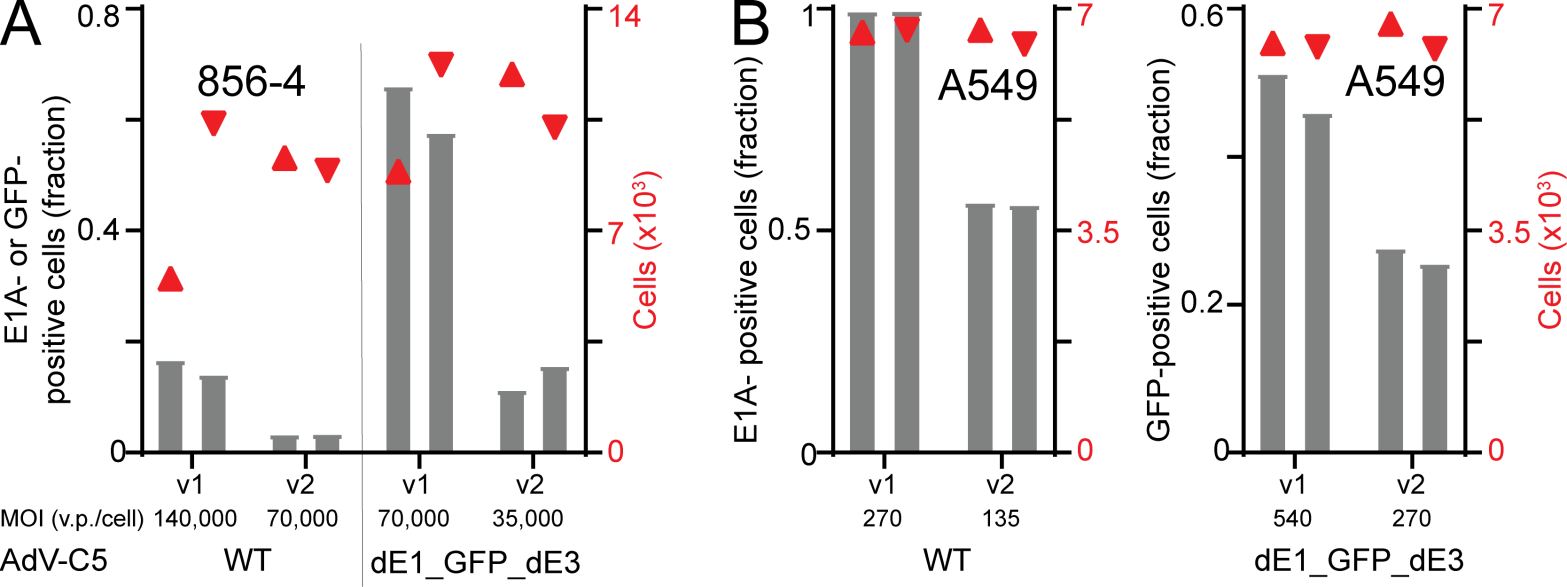
AdV-C5 poorly infects hiPSC-derived macrophages. (**A**) Macrophages of clone 856-4 were inoculated with WT-AdV-C5 or a replication-deficient AdV-C5_dE1_GFP_dE3 virus (dE1_GFP_dE3B) expressing GFP from the CMV major immediate-early e/p and analyzed for infection by E1A immunostaining (WT) or GFP fluorescence 31.5 h pi using wide-field microscopy. (**B**) Both WT and dE1_GFP_dE3 viruses efficiently infect A549 cells, analyzed 24 h pi, as in panel A. The graphs show the fraction of E1A- or GFP-positive cells, as well as the total number of cells analyzed under given multiplicity of infection (MOI; number of physical input v.p./cell) and input virus dilutions (V1, V2). Red triangles indicate overall cell counts. Two technical replicates are shown separately.

To characterize AdV-C5 infection of hiPSC-derived macrophages and ensure reproducibility, we used five different hiPSC lines (854-2, 840-3, 856-4, DW4, WTB6) engineered from healthy individuals and followed established protocols for macrophage differentiation (41–43). The macrophages expressed the key surface markers CD11b, CD14, CD16, CD163, and CD45 (41–43). We first tested the transduction efficiency by incubating the 856-4 macrophages with wild-type (WT) AdV-C5 or a non-replicating vector AdV-C5_dE1_GFP_dE3, lacking the E1 and E3 transcription units and expressing enhanced green fluorescent protein (GFP) from a cytomegalovirus major immediate-early enhancer/promoter (CMV-e/p). Surprisingly, WT yielded very low numbers of infected cells, as indicated by immunostaining of E1A, whereas the CMV-e/p-driven reporter vector gave robust GFP expression 31.5 h post-infection (pi) at comparable input physical particle numbers (Fig. 1A). The poor E1A expression from AdV-C5 was observed also in macrophages derived from the other hiPSC lines (see Fig. 4 and 5; Fig. S1 and S2), while AdV-C5 transduced human lung carcinoma A549 cells even more efficiently than AdV-C5_dE1_GFP_dE3, and significantly lower input virus amounts were required for A549 transduction (Fig. 1B). These data show that the WT or the CMV-e/p reporter virus transduction of hiPSC-derived macrophages was of low efficiency in comparison to A549 cells, requiring about 130-fold more inoculum than A549 cells.

### Attenuated AdV-C5 entry into hiPSC-derived macrophages

**Fig 2:**
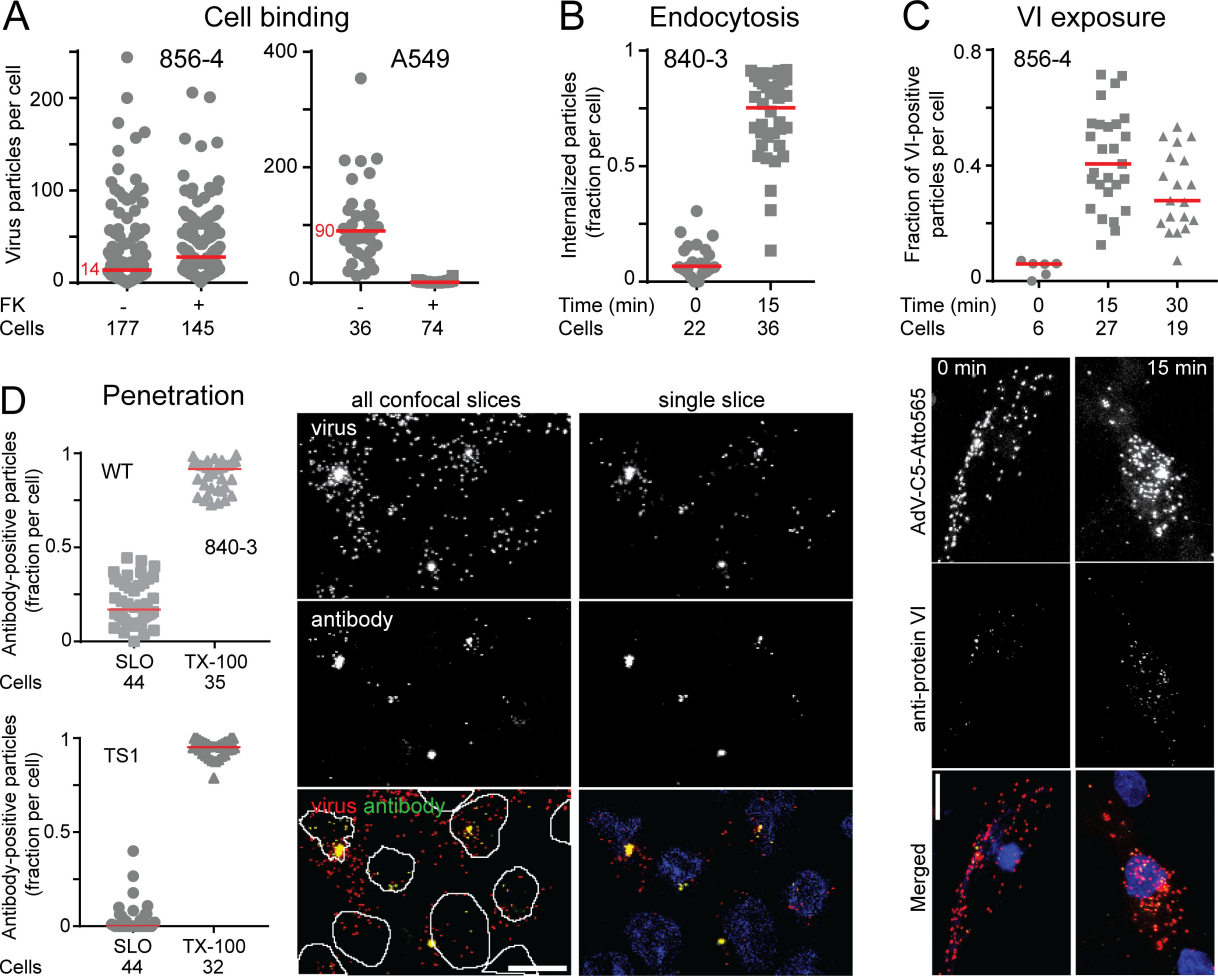
AdV-C5 entry into hiPSC-derived macrophages is impeded at cell binding and penetration. (**A**) Soluble fiber knob (FK) does not inhibit binding of AdV-C5 to hiPSC-derived macrophages. 856-4 macrophages or A549 cells were preincubated with FK for 30 min on ice, inoculated with Atto 565-labeled AdV-C5 at MOI ~350,000 and ~117,000 v.p./cell, respectively, in the presence of FK for 60 min on ice, and analyzed for cell-associated viruses by maximum-projection confocal microscopy. The graph shows the number of v.p./cell. One dot represents one cell, and horizontal red bars represent median values. The number of cells analyzed is indicated. (**B**) Efficient uptake of AdV-C5 into hiPSC-derived macrophages. Atto 565-labeled AdV-C5 was incubated with 840-3 cells at 4°C for 60 min at MOI ~86,000 v.p./cell, washed, and either kept at 4°C or shifted to 37°C for 15 min. 9C12 anti-hexon antibodies at 4°C tagged surface-associated particles prior to fixation and staining with secondary Alexa Fluor 488-conjugated antibodies. Particles devoid of 9C12 signal were scored as internalized, where one dot represents one cell. Only cells that had ≥5 v.p./cell were scored. Horizontal bars represent median values, and the number of cells analyzed is indicated. Nuclei were stained with DAPI, cell area with Alexa Fluor 647 NHS-ester, and samples were imaged by confocal microscopy. (**C**) Particles entering into hiPSC-derived macrophages expose the membrane-lytic protein VI. Atto 565-labeled AdV-C5 (MOI ~62,000 v.p./cell) were cold-bound to 856-4 cells as described in panel B, and particle-associated protein VI signal was determined after 15 min and 30 min shift to 37°C by staining fixed and permeabilized samples with anti-protein VI and secondary Alexa Fluor 488-conjugated antibodies. Control cells were kept at 4°C. The graph shows the fraction of VI-positive v.p./cell, with one dot representing one cell. Horizontal bars represent median values, and the number of cells analyzed is indicated. Representative confocal images are shown. Scale bar = 10 µm. (**D**) Poor endosomal penetration of AdV-C5. Atto 565-labeled WT-AdV-C5 (MOI ~21,000 v.p./cell) and the penetration-deficient TS1-AdV-C2 labeled with Alexa Fluor 488 were added to 840-3 cells at 37°C for 60 min. Samples were washed and returned to 37°C for 65 min. The plasma membrane was then perforated with streptolysin O (SLO) followed by tagging of cytoplasmic particles with antibodies (9C12 specific for AdV-C5 hexon or anti-Alexa Fluor 488 to detect TS1). Endosomal membranes remained impermeable to antibodies. After fixation and detergent permeabilization, WT and TS1 tagged with primary antibodies were visualized by secondary Alexa Fluor 488- or Alexa Fluor 594-conjugated antibodies, respectively. Control cells (TX-100) were fixed without SLO treatment, permeabilized with Triton X-100, and immunostained as the SLO samples. Samples were imaged by confocal microscopy. The graph shows the fraction of antibody-positive virus particles per cell, with one dot representing one cell. Horizontal bars represent median values. Cells with ≥5 viruses per cell were analyzed in the indicated number of cells. Micrographs are from SLO-treated WT samples (maximum projection of confocal stacks or a single Z-stack slice). The WT-inoculated cells contained prominent cytosolic clusters of AdV-C5 located in the perinuclear area, as indicated by DAPI nuclear stain and nuclear outlines from maximum-projection overlay images. Nucleus from a single Z-stack slice is shown in blue. Scale bar = 10 µm.

To determine the mechanism underlying the low AdV-C5 transduction in hiPSC-derived macrophages, we analyzed AdV-C5 entry into these cells and first used Atto565-labeled virus particles (v.p.) to score attachment of AdV-C5 to 856-4 macrophages. Previous studies have shown that the fluorophore tag on AdV particles does not interfere with virus interactions with epithelial or immune cells (44, 45). Confocal microscopy and single-particle measurements indicated that AdV-C5 attachment to macrophages was highly variable between individual cells, and, on average, about 6-fold less effective than attachment to A549 cells at 3-fold lower input virus amounts, yet unaffected by soluble AdV-C5 fiber knobs, unlike attachment to A549 cells (Fig. 2A). AdV-C5 binds to the high-affinity receptor coxsackievirus AdV receptor (CAR) on A549 cells through the distal knob of the fiber protein (reviewed in reference 46), but CAR is not expressed at significant levels on macrophages, including the hiPSC-derived macrophages (36, 42, 45) (see Dataset A1 at https://doi.org/10.5281/zenodo.18656599). The hiPSC-derived macrophages lack an abundant or high-affinity receptor for AdV-C5 (Fig. 2A), but the identity of the receptor(s) mediating AdV-C5 infection was not investigated in this study.

To address steps downstream of receptor binding, we used single-cell, single-particle assays developed in our laboratory to quantify intracellular transport of incoming AdV-C5 to the nucleus (44, 47, 48). The internalization of AdV-C5 into 840-3 macrophages was tested by an immunoassay scoring, in intact cells, the number of Atto565-tagged particles accessible to the 9C12 anti-hexon antibody, i.e., the particles remaining at the cell surface, and the antibody-free internalized particles. As shown in Fig. 2B, virus uptake occurred with >50% efficiency in the majority of the cells within 15 min of warm incubation, whereas particles remained largely accessible to the anti-hexon antibodies in control cells kept at +4°C. Thus, the unknown receptor(s) mediate efficient virus uptake in these cells.

Virus penetration into the cytoplasm requires externalization of the viral membrane-lytic protein VI from the capsid interior and VI-mediated disruption of the limiting endosomal membrane (44, 47, 49–51). As shown in Fig. 2C, a transient increase in the fraction of VI-positive viruses was observed upon shifting 856-4 cells to 37°C, whereas particles in control cells kept at +4°C were largely devoid of protein VI signal. Overall, the protein VI exposure occurred in the hiPSC-derived macrophages with a roughly similar efficiency as in A549 cells (52).

**Fig 3:**
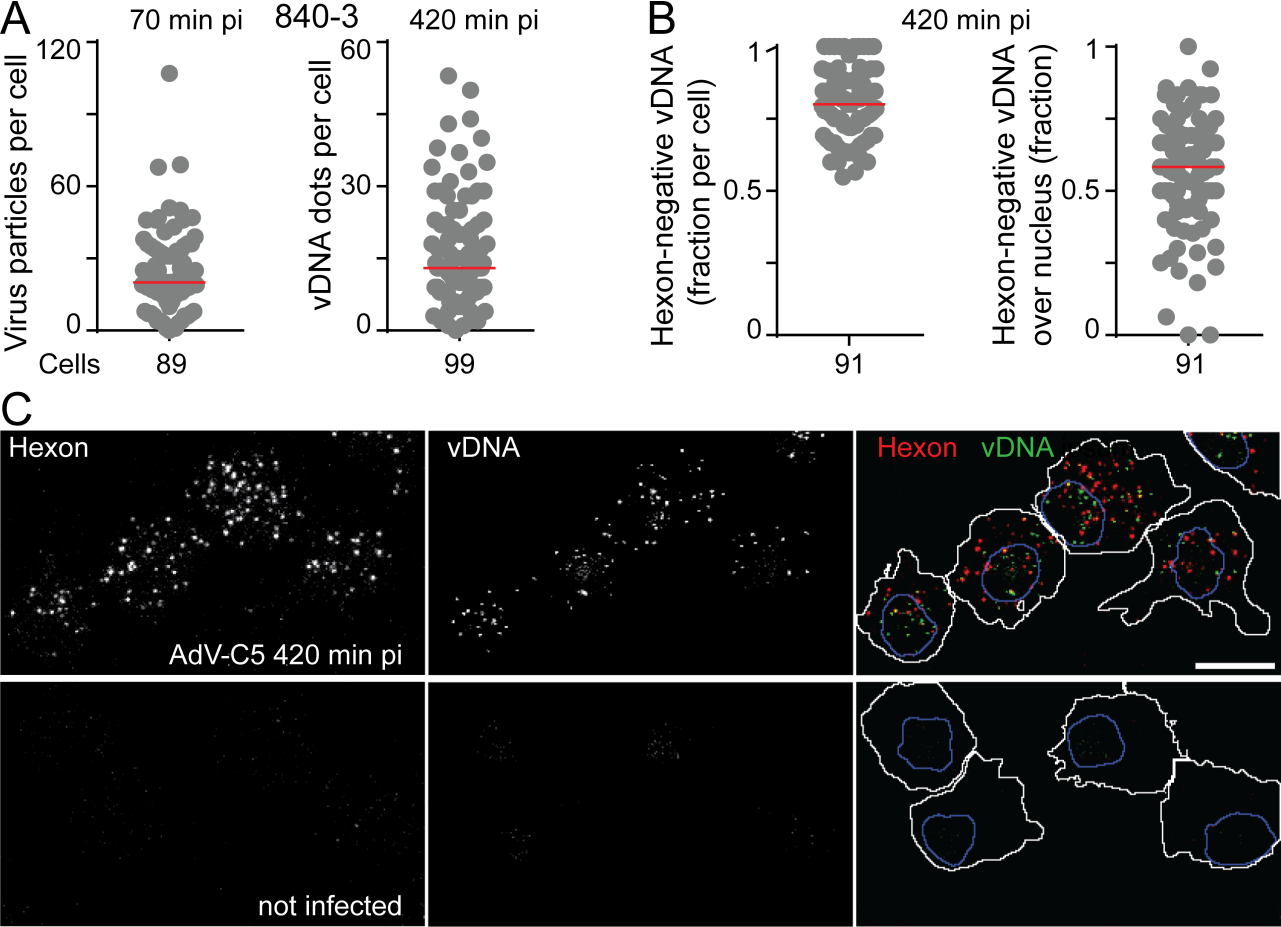
Uncoating of vDNA of incoming viral DNA in hiPSC-derived macrophages and substantial cytoplasmic misdelivery. 840-3 macrophages were inoculated with EdC-labeled AdV-C5 at MOI ~12,500 v.p./cell for 60 min, washed, and incubated to the indicated time point at 37°C. Virus particles were visualized in fixed cells by 9C12 anti-hexon and secondary Alexa Fluor 488- or Alexa Fluor 594-conjugated antibodies, whereas vDNA was visualized by Alexa Fluor 488-conjugated azide, and analyzed by confocal fluorescence microscopy. (**A**) Comparison of cell-associated single virus (70 min pi) and single vDNA counts (420 min pi) shows large cell-to-cell variability and a time-dependent reduction in cell-associated vDNA with median values of 20 and 13 for virus and vDNA, respectively. One dot represents one cell. Horizontal bars represent median values. The number of cells analyzed is shown. (**B**) Efficient uncoating (>50%) of incoming vDNA, measured by separation of vDNA from hexon-stained virus capsid 420 min pi. The majority of cells have >30% of the uncoated vDNA misdelivered to the cytoplasm. One dot in the graphs represents one cell, and only cells with ≥5 vDNA dots were analyzed. Horizontal bars represent median values. (**C**) Representative images 420 min pi (maximum projections of confocal stacks). The vDNA images were uniformly scaled to fade the background signal and boost the vDNA signal. Nucleus and cell area are indicated in the overlay figure. Scale bar = 10 µm.

We next measured AdV-C5 penetration into the cytoplasm of 840-3 cells using atto565-tagged viruses and streptolysin O (SLO) permeabilization to perfuse 9C12 anti-hexon antibodies into the cells (44). Antibody-positive v.p. were observed in SLO-permeabilized cells (Fig. 2D). These particles represented cytoplasmic particles, since penetration-deficient Alexa Fluor 488-conjugated AdV-C2-TS1 particles (53) were largely inaccessible to anti-Alexa Fluor 488 antibodies. TS1 particles contain immature protein VI, are highly stable, do not expose the membrane-lytic protein VI, and are trapped in endosomes (47, 53, 54). Control cells after Triton X-100 permeabilization contained immunostained WT or TS1 particles. The penetration of AdV-C5 in hiPSC-derived macrophages was less efficient than in A549 cells (44). In addition, the macrophages exhibited a transient perinuclear clustering of cytosolic WT particles at 125 min pi (Fig. 2D), which was dispersed at 420 min pi (see Fig. 3C). This phenotype was strikingly similar to centrosomal clustering of AdV-C particles in cells treated with a nuclear export inhibitor leptomycin B (55), but the exact nature of the phenotype remains to be characterized.

Cytoplasmic AdV particles translocate to the nucleus, and the viral capsid undergoes disassembly at the cytoplasmic side of the nuclear pore complex, freeing vDNA for nuclear import (52, 56–59). We next analyzed this uncoating step using EdC-tagged vDNA (48). The median number of cell-associated capsids stained with 9C12 was 20 per cell at 70 min pi, a time point where uncoating is about to start (Fig. 3A). The vDNA puncta had a median of 13 at 420 min pi, suggesting that incoming vDNA decayed in the course of infection, albeit similar as reported in A549 cells (44). vDNA uncoating was efficient as indicated by >50% of vDNA puncta lacking hexon signal at 420 min pi (Fig. 3B and C). Remarkably, the majority of cells misdelivered at least 30% of the uncoated vDNA to the cytoplasm (Fig. 3B, right panel), a feature also observed in epithelial cells with high cell-to-cell variability (45, 52, 60). Taken together, the results demonstrate that macrophage invasion by AdV-C5 is attenuated at select entry steps compared to A549 cells, particularly cell binding and endosomal penetration. Yet, there is no evidence for a single, insurmountable restriction point in AdV-C5 entry into hiPSC-derived macrophages.

### Low activity of the E1A enhancer/promoter in macrophages, independent of IFN-mediated restriction

**Fig 4:**
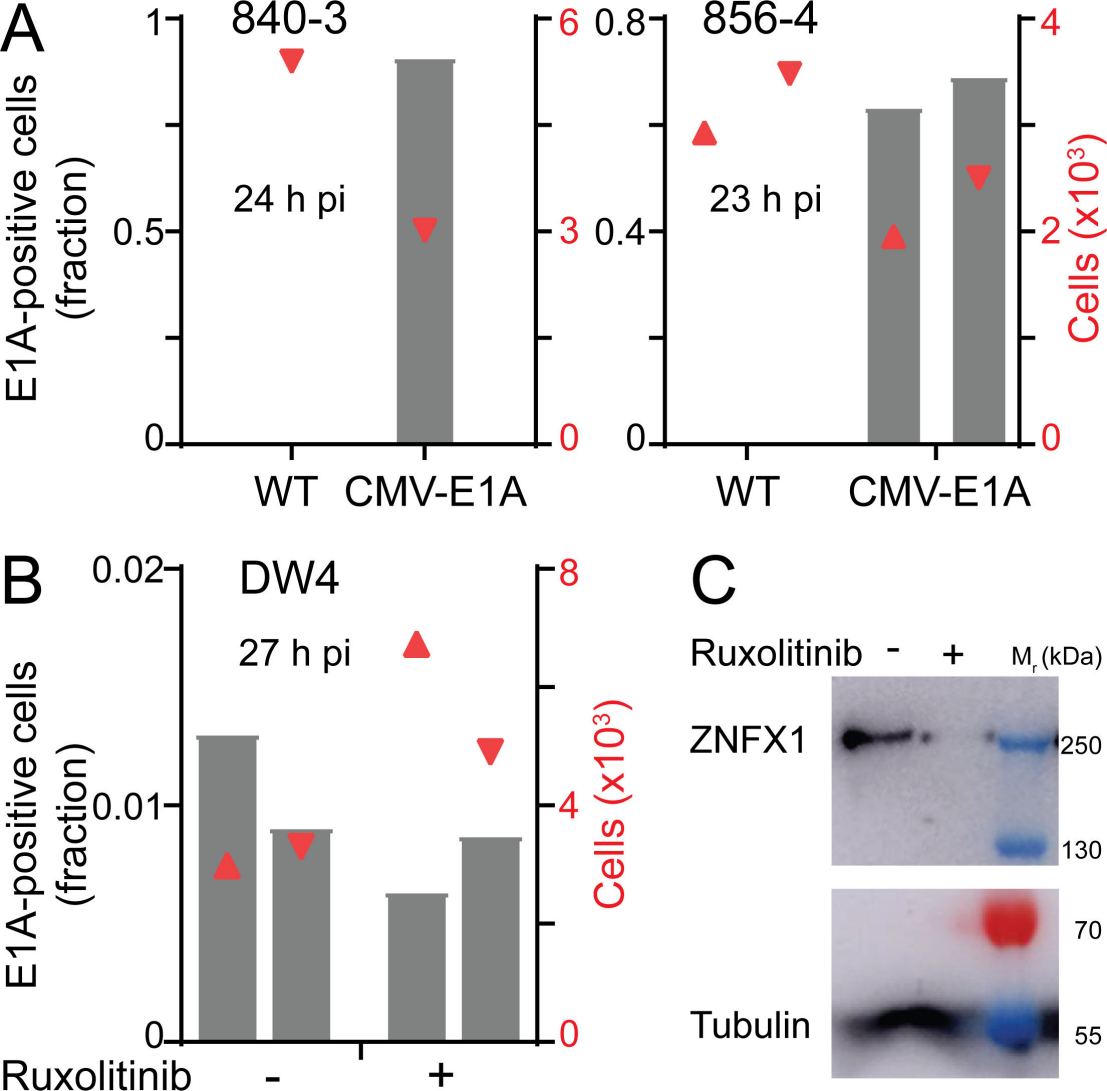
IFN signaling-independent restriction of the E1A enhancer/promoter in hiPSC-derived macrophages. (**A**) Replacement of native E1A-e/p by a CMV-e/p dramatically boosts the E1A expression in hiPSC-derived macrophages. 856-4 or 840-3 cells were incubated with similar input virus amounts of AdV-C5 (WT) and AdV-C5-CMV-E1A (CMV-E1A) at MOI ~3,500 v.p./cell for 15 h (856-4) or 9.5 h (840-3). Unbound virus was then removed, and incubation was continued for an additional 8 h (856-4) or 14.5 h (840-3). E1A expression levels were analyzed by anti-E1A and secondary fluorophore-conjugated antibody stainings. Nuclear E1A signal was determined by wide-field microscopy, and shown are fractions of E1A-positive cells. The two technical replicates in the 856-4 experiment are shown separately. (**B**) Poor activity of the native E1A-e/p in hiPSC-derived macrophages is unlikely to be due to IFN-mediated repression. DW4 cells were pretreated with the JAK1/2 inhibitor ruxolitinib (2 µM) over two nights prior to and during inoculation of WT at MOI ~13,100 v.p./cell for 7.5 h. Cells were analyzed 27 h pi as in panel A. (**C**) Western blot analyses of cells pretreated with 2 µM ruxolitinib for 3 h, followed by IFNα2a for an additional 26.5 h, showing the ISG ZNFX1 and alpha/beta tubulin as a loading control stained with HRP-conjugated secondary antibodies. Relative molecular weight in kDa.

The poor transduction by AdV-C5 compared to the CMV-GFP reporter AdV-C5 along with the presence of incoming vDNA in the nucleus suggested that the E1A-e/p activity was restricted in hiPSC-derived macrophages. To control for possible differences in stability of E1A protein and GFP, we compared E1A expression from the native E1A-e/p with that from the CMV immediate-early e/p, using WT and AdV-C5-in340-Δ2-CMV-E1A (AdV-C5-CMV-E1A), the latter lacking the E1B transcription unit (27). The CMV-e/p was already found to be active in our hiPSC-derived macrophages, in accordance with reports in monocyte-derived macrophages (61). We infected 856-4 and 840-3 cells with WT or CMV-E1A viruses at similar multiplicity of infection (MOI), followed by immunostaining of E1A. This revealed that the replacement of the native E1A-e/p with the CMV-e/p greatly boosted E1A expression (Fig. 4A). Single-cell and single-molecule RNA-FISH experiments in 840-3 and 854-2 cells indicated that this difference in E1A protein levels was due to significantly higher E1A transcript counts in AdV-C5-CMV-E1A-infected cells than in WT infection (Fig. S1A).

We tested if the E1A-e/p suppression was due to IFN type 1 or 2 signaling. The inhibition of the Janus kinase (JAK)1/2 and downstream signal transducer and activator of transcription (STAT) pathways by ruxolitinib (62) did not boost E1A protein expression in WT-infected DW4 macrophages or E1A transcripts in WT-infected WTB6 macrophages (Fig. 4B; Fig. S1B), but inhibited IFNα2a-mediated induction of ZNFX1 (zinc finger NFX1-type containing 1), an IFN-stimulated gene (ISG, 63) (Fig. 4C). Thus, IFN-signaling is not the main reason for poor activity of E1A-e/p in hiPSC-derived macrophages.

### E1A expression in *trans* boosts native E1A enhancer/promoter

**Fig 5:**
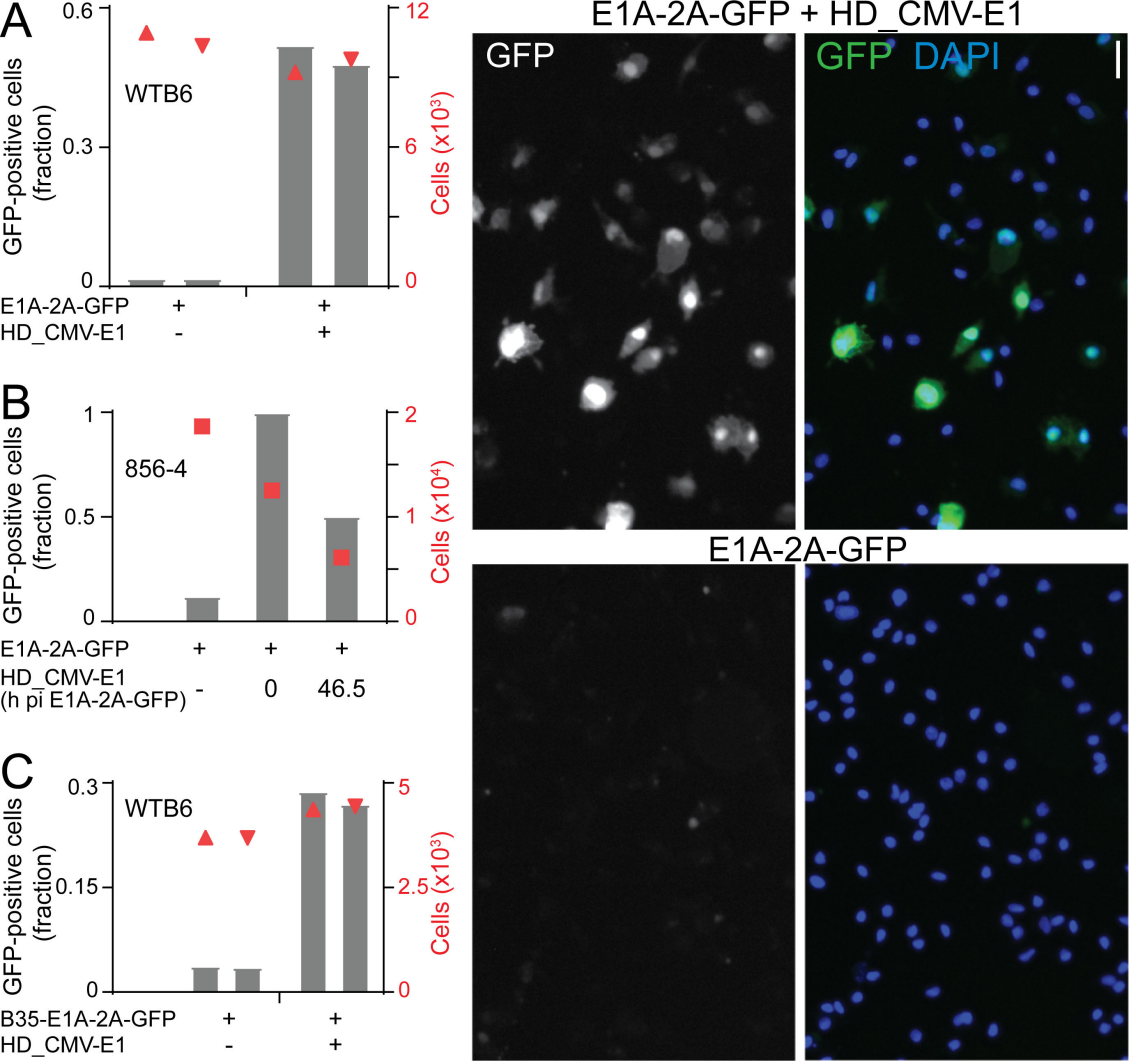
CMV-e/p-driven expression of E1A transactivates the native E1A-e/p and allows for productive AdV-C5 infection of hiPSC-derived macrophages. (**A**) WTB6 macrophages were inoculated with AdV-C5-E1A-2A-GFP (E1A-2A-GFP, native E1A-e/p), or with E1A-2A-GFP plus HD_CMV-E1 at a total MOI of ~25,500 v.p./cell for 7 h. The activity of the native E1A-e/p was scored by analyzing the fraction of GFP-positive cells by wide-field microscopy 30 h pi. Two technical replicates are shown, including the number of analyzed cells, as well as representative images on the right-hand side. Scale bar = 50 µm. (**B**) GFP expression from E1A-2A-GFP induced by superinfecting HD_CMV-E1 46.5 h post-inoculation of E1A-2A-GFP, indicating persistence of infectious vDNA. 856-4 macrophages were inoculated with AdV-C5-E1A-2A-GFP for 6 h (MOI ~51,000 v.p./cell); 46.5 h later, with HD_CMV-E1 for 6 h, and analyzed 74.5 h after initial E1A-2A-GFP infection. 0 h indicates a co-inoculation of E1A-2A-GFP plus HD_CMV-E1, analyzed at 28 h pi. (**C**) Coinfection of WTB6 macrophages with AdV-B35-E1A-2A-GFP (B35-E1A-2A-GFP) plus HD_CMV-E1 boosts the activity of native AdV-B35 E1A-e/p. Cells were incubated with the B35-E1A-2A-GFP virus alone (MOI ~12,000 v.p./cell) or together with the HD_CMV-E1 virus for 7 h, and the fraction of GFP-positive cells was determined 30 h pi. Two technical replicates are shown.

The E1A-e/p and other AdV transcription units are positively regulated by the 289-residue 13S E1A protein (64–66). We tested whether E1A expressed from CMV-e/p could transactivate E1A expression from the native E1A-e/p in hiPSC-derived macrophages. The cells were co-inoculated with a gutless, helper-dependent vector, AdV-C5_CMV-E1 (HD_CMV-E1), and AdV-C5-E1A-2A-GFP for 7 h. HD_CMV-E1 contains the E1A unit under the CMV immediate-early e/p and the E1B transcription unit under its native promoter and lacks all other viral coding sequences. AdV-C5-E1A-2A-GFP expresses GFP from the native E1A-e/p as a separate protein from E1A owing to cotranslational peptide splitting and reinitiation (ribosome-skipping by 2A) (67). Coinfection significantly boosted the number of GFP-expressing WTB6 macrophages compared to single AdV-C5-2A-GFP infection at 30 h pi, akin to other macrophage lines (Fig. 5A and B; Fig. S2). A boost in GFP-positive cells was also obtained if the transactivating vector was added 2 days after the AdV-C5-E1A-2A-GFP infection, as shown for 856-4 macrophages, albeit to a lesser extent than in simultaneous infections (Fig. 5B). Remarkably, a B-species AdV, AdV-B35-E1A-2A-GFP, also exhibited low E1A-e/p activity in hiPSC-derived macrophages, which was boosted by coinfection with the gutless HD_CMV-E1 vector (Fig. 5C).

### Bulk RNA-seq analyses of AdV-C5-infected hiPSC-derived macrophages

**Fig 6:**
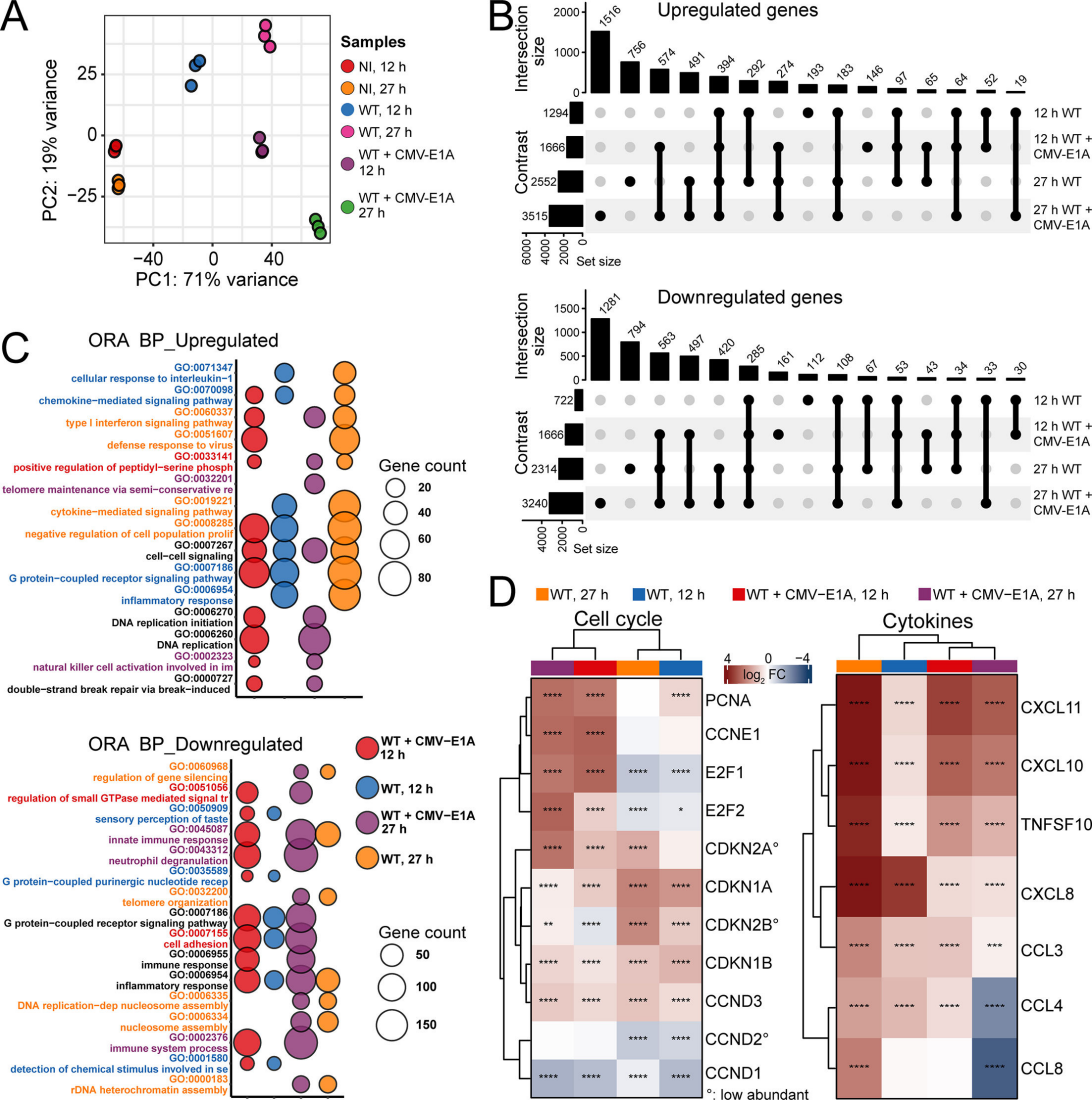
Bulk RNA-seq analyses of hiPSC-derived macrophages upon single WT or WT plus CMV-E1A coinfection. Macrophages (856-4) were inoculated with single WT-AdV-C5 or WT plus AdV-C5-CMV-E1A (CMV-E1A) at an MOI of ~42,000 v.p./cell for 12 h. After removal of unbound virus, samples were further incubated for 0 h or 15 h and then subjected to bulk RNA-seq analyses of total RNA depleted of ribosomal RNA. Non-infected cells from both time points served as controls. All samples had three replicates. The PCA plot in panel **A** shows the distinctness of the sample profiles and low technical variability indicated by the clustering of the corresponding replicates. (**B**) “UpSet” plot of differentially expressed genes across the different samples, |log2 fold change| > 1 and FDR-adjusted *P*-value threshold 0.05. (**C**) Over-representation analyses (ORA) of top five up- and downregulated biological process (BP) GO terms based on *P*-value. The dot size indicates the number of differentially expressed genes in a particular GO term. GO terms are indicated on the left and color-coded according to the sample. Black color indicates that the particular GO term was in the top five terms of more than one sample. (**D**) Tile plots of differentially expressed genes (DEG) showing selected G1/S-phase cell cycle regulators (left), and the most abundant upregulated cytokine transcripts compared to non-infected samples (right; log2 fold change > 1 and FPKM > 300). Color code indicates log2 fold change (log2 FC). Genes not differentially expressed are shown as white boxes. The *P*-values of DEG are indicated by stars (*P < 0.05, **P < 0.01, ***P < 0.001, ****P < 0.0001), and low abundant transcripts by degree (°).

The transcriptional landscape is a powerful descriptor of the cell state. We generated comparable transcriptomes of clone 856-4 macrophages at 12 and 27 h pi, comprising single WT and WT plus AdV-C5-CMV-E1A helper virus coinfections and non-infected controls. We did not test single AdV-C5-CMV-E1A infections because this virus lacks the anti-apoptotic functions of E1B (27), and infection leads to premature cell death. Principal component analysis (PCA) showed that PC1 explained 71% of the variance between samples, and PC2 explained 19% (Fig. 6A). In PC1/PC2 plots, the sample replicates robustly clustered together, while the non-infected and the coinfected 27 h samples were separated furthest from each other along the PC1 axis.

Pairwise comparison of infected and non-infected samples using edgeR identified differentially expressed genes (DEGs; see Dataset A1 at https://doi.org/10.5281/zenodo.18656599). For example, thehost transcriptome was changed to a larger extent at 27 h pi than at 12 h pi (|log2 fold change| > 1 and FDR-adjusted *P*-value threshold 0.05), as shown in “UpSet” plots (Fig. 6B). Strikingly, each sample had both unique and shared DEGs, the latter amounting to 394 upregulated and 285 downregulated genes in all infected samples. The upregulated DEGs included 36 viral genes. Over-representation analyses (ORA, based on *P*-value) of the top five biological process (BP) gene ontology (GO) terms are shown in Fig. 6C. The corresponding DEGs are listed in Dataset A2 at https://doi.org/10.5281/zenodo.18338933.

Top BPs upregulated in single WT infection samples at both 12 h and 27 h pi were dominated by terms linked to cell signaling and host defense, including upregulation of cytokine transcripts, the most abundant ones at 12 h pi being CXCL8, CCL3, and CCL4 with log2 fold change >1 and fragments per kilobase of transcript per million mapped reads (FPKM) >300 compared to non-infected cells, whereas CXCL11, CXCL10, CXCL8, CCL3, CCL4, and CCL8 chemokine transcripts were strongly increased at 27 h pi along with TNFSF10 (log2 fold changes >1.6 and FPKM >700; Fig. 6D). The inflammatory response (GO:0006954) and G protein-coupled receptor (GPCR) signaling pathway (GO:0007186) were among the top five terms in both the up- and downregulated BPs in the 12-h WT single infection samples. Upregulated inflammatory response DEGs included cytokines CSF1, CXCL8, TNF, CCL3, CCL4, and CCL3L3, whereas downregulated inflammatory response DEGs were linked to host response to bacterial infection (CD14, STAB1), TGF-beta signaling (NRROS, FOS), or suppression of inflammatory responses (TMIGD3, CD163). In the case of GPCR signaling, 40% of the upregulated DEGs were also classified as upregulated inflammatory response genes, predominantly cytokines, and likely reflect an *in vivo* situation in which macrophages coordinate host defense with other cell types. Top downregulated BPs in the 27 h pi WT single infection sample included GO terms nucleosome assembly, telomere organization, rDNA heterochromatin assembly, DNA replication-dependent nucleosome assembly, and regulation of gene silencing.

In coinfections, on the other hand, top upregulated BPs included terms linked to DNA replication, cell-cell signaling, and positive regulation of peptidyl-serine phosphorylation of STAT protein. The GO terms linked to DNA replication indicated cell cycle changes, also seen in the upregulation of transcripts for transcription factors driving S-phase transition (E2F1, E2F2), cyclin E (CCNE1), and the S-phase marker PCNA, along with low-abundance cell cycle transcripts (Fig. 6D, left panel). Although top downregulated BPs in coinfections at both time points were dominated by host defense responses, the coinfection abundantly induced CXCL11, CXCL10, and CCL3 transcripts at 12 h pi (log2 fold change >1 and FPKM counts >300), while at 27 h pi, only CXCL10 (log2 fold change >2.3, FPKM ~1,000) remained strongly induced (Fig. 6D, right panel).

Two host transcripts, CXCL8 and IFIT2, were strongly induced in the WT single infections of 856-4 macrophages, whereas IFIT2 was only strongly induced in the coinfections (RNA-seq data in Dataset A1 at https://doi.org/10.5281/zenodo.18656599). RT-qPCR crosstests, however, indicated no induction for CXCL8 in infected 854-2 macrophages, and IFIT2 induction appeared to occur less efficiently than in 856-4 cells (Fig. S3A). Cytokine quantification in the supernatant of 856-4 cells confirmed increased CXCL8 at 12 and 27 h pi in the single WT infection (Fig. S3B). Basal CXCL8 levels in the non-infected 854-2 cells were relatively high (~2.5 ng/mL), comparable to induced secretion from 856-4 cells, and did not increase upon infection (Fig. S3B; also see Dataset A3 at https://doi.org/10.5281/zenodo.18338933). The RNA-seq data suggested robust induction of IFN transcripts in AdV-C5 infections, but the overall transcript counts were low (see Dataset A1), suggesting that IFN transcripts were probably induced only in a low number of cells. Accordingly, only low levels of IFN (<70 pg/mL) were observed in the culture supernatants of infected cells (Fig. S3B; also see Dataset A3). Collectively, these data show that single- and co-infections give rise to distinct cell states favoring DNA replication, cell cycle, or innate immune response, notably with hiPSC-clone-dependent cytokine expression profiles.

### Repression of the E1A enhancer/promoter is a major barrier for productive AdV-C5 infection

**Fig 7:**
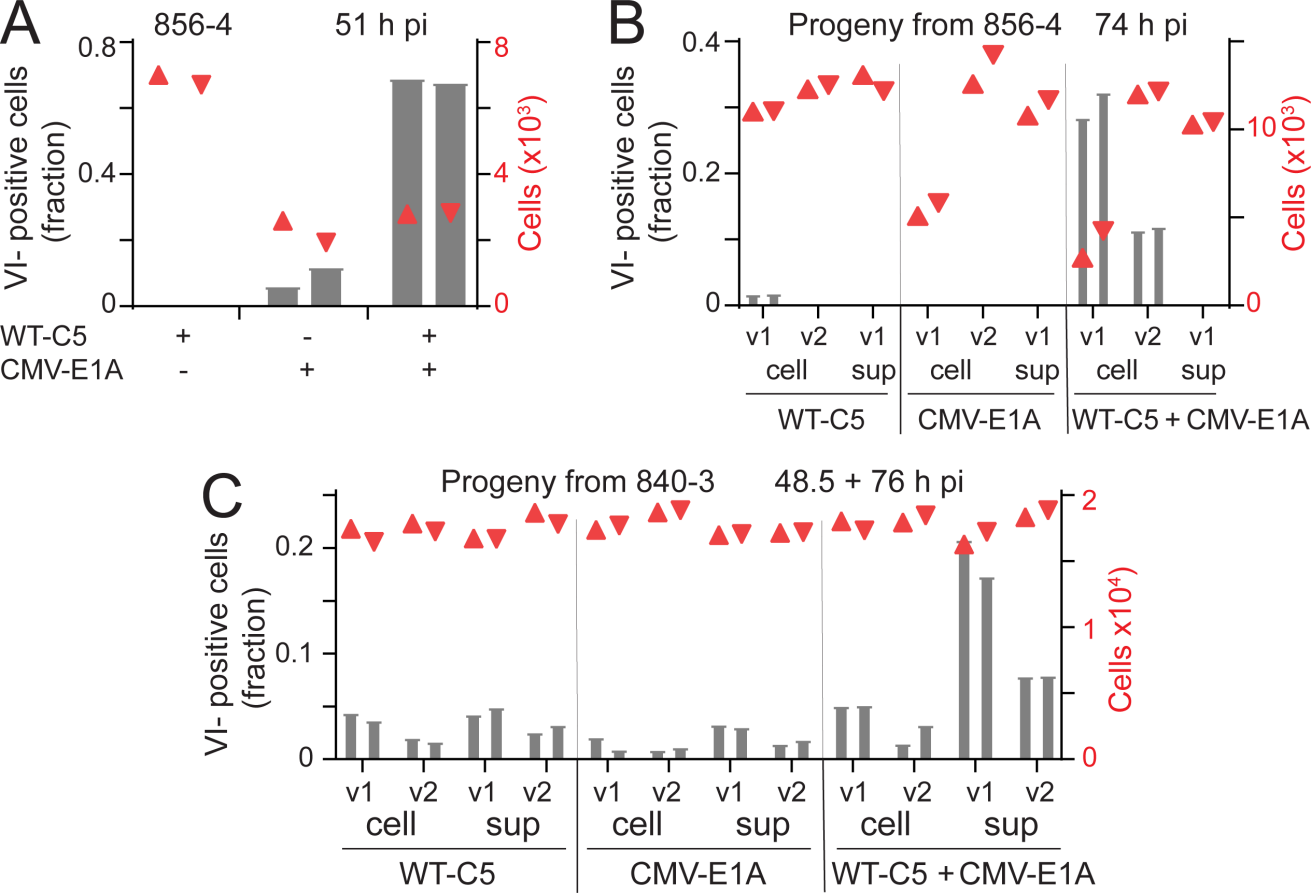
CMV-e/p-driven expression of E1A allows for productive AdV-C5 infection of hiPSC-derived macrophages. (**A**) AdV-C5 infection proceeds to the late phase in 856-4 macrophages coinfected with AdV-C5 and AdV-C5-in340-Δ2-CMV-E1A (CMV-E1A). Cells were inoculated with WT or CMV-E1A alone or together at a total MOI of ~42,000 v.p./cell for 8.5 h, and analyzed by immunostaining using antibodies against the late viral protein VI at 51 h pi. Two technical replicates are shown. (**B**) WT and CMV-E1A coinfection of 856-4 macrophages leads to progeny production. Cell- and medium-associated (supernatant, sup) progeny were collected at 74 h pi and titrated on A549 cells by immunostaining for the late viral protein VI at 32 h pi, with v1 and v2 representing 2-fold equivalent dilutions of cell or supernatant material. Two technical replicates of the samples are shown. (**C**) Boosting a dormant WT AdV-C5. 840-3 macrophages were incubated with WT at an MOI of ~42,000 v.p./cell for 9 h, washed, and inoculated for 7 h with CMV-E1A at 48.5 h pi. Single WT and CMV-E1A inoculations served as controls. Cell- and supernatant-progeny were collected 75 h post-superinfection and titrated on HeLa cells by immunostaining for the late viral protein VI 39 h pi, with v1 and v2 representing 2-fold equivalent dilutions of cell or supernatant material. Two technical replicates of the samples are shown.

E1A proteins transactivate other AdV transcription units and remodel the host gene expression, enabling progression of the host cell into S-phase and virus replication (19, 23, 65). Indications for a change toward an S-phase-like cell cycle stage in the WT plus AdV-C5-CMV-E1A helper virus coinfections could be seen in the RNA-seq data (Fig. 6; also see Dataset A1 at https://doi.org/10.5281/zenodo.18656599). To test if hiPSC-derived macrophages became permissive for late-phase AdV infection and progeny formation in the coinfection setting, we first analyzed late viral protein VI expression at 51 h pi by immunofluorescence. Coinfection yielded a high number of VI-positive cells, in contrast to single infections (Fig. 7A). As noted above, AdV-C5-CMV-E1A is a replication-impaired virus because it lacks the early E1B transcription unit, which encodes proteins that suppress untimely apoptotic death of infected cells and proteins that contribute to neutralization of host inhibitors of virus replication (68).

We next analyzed progeny production from WT- and AdV-C5-CMV-E1A-coinfected 856-4 cells at 74 h pi by titration in A549 cells using anti-VI immunostaining. The cell extracts and culture supernatants from single infections yielded essentially no VI-positive cells, indicating low or negligent progeny production from these infections (Fig. 7B). In contrast, a significantly higher number of infectious progeny virus was produced from the WT and AdV-C5-CMV-E1A coinfections, but the progeny particles remained cell-associated at 74 h pi. HD_CMV-E1 superinfection activated E1A expression from dormant AdV-C5-E1A-2A-GFP (see Fig. 5B). We finally tested whether delayed superinfection would trigger viral progeny production from dormant WT AdV-C5. The superinfecting AdV-C5-CMV-E1A, added with a delay of 48.5 h, increased the WT titer in the supernatant of 840-3 macrophages at 75 h post-addition, as indicated by progeny titration on HeLa cells (Fig. 7C). Control samples collected from single WT or CMV-E1A infections at 123.5 h and 75 h pi, respectively, gave reduced titers. Taken together, the results indicate that the poor activity of E1A-e/p is a major block to productive AdV-C5 infection of hiPSC-derived macrophages.

## DISCUSSION

Macrophages function in homeostatic tissue repair and acute innate defense against pathogens, acting as both sensors and effectors in the tissue microenvironment (69, 70). Macrophages tend to resist immune clearance and act as reservoirs for HIV, herpesviruses, SARS-CoV-2, or rubella (4–7). The current study overcomes the limited availability of primary human macrophages and shows that hiPSC-derived macrophages restrict AdV infection at multiple levels, and dormant vDNA in these cells can be triggered to support productive infection.

### AdV entry restriction

Although early work had shown that AdV can enter into macrophages, virus replication does not efficiently occur in these cells (33). Our study in hiPSC-derived macrophages identifies three major restriction points before viral replication: virus attachment to cells, endosomal penetration of incoming virus, and immediate-early viral gene expression in the nucleus. hiPSC-derived macrophages lack an abundant high-affinity receptor and take up less AdV-C5 than epithelial lung carcinoma A549 cells, notably in a fiber-knob–independent manner. The human ortholog of the mouse macrophage receptor for AdV-C5, SR-A6, is unlikely to be a receptor for AdV-C5 on hiPSC-derived macrophages, since these cells express only low levels of SR-A6 (42) (see Dataset A1 at https://doi.org/10.5281/zenodo.18656599), and human SR-A6 binds AdV-C5 less efficiently than the mouse ortholog (45). Integrin αMβ2 would be one receptor candidate, as both of its subunits are expressed in hiPSC-derived macrophages (see Dataset A1), and it mediates fiber-independent attachment of AdV-C2 to human THP-1 monocytic leukemia cells (71).

Uptake of bound AdV-C5 into hiPSC-derived macrophages was efficient, but endosomal escape was inefficient compared to A549 cells, although the particles externalized the membrane-lytic protein VI from their interior, a prerequisite for virus escape from endosomes. It is possible that protein VI binding to the limiting endosomal membrane is inefficient due to a lack of sufficient ceramide lipids in macrophages, which become enriched in the plasma membrane and endosomes upon lysosomal secretion of acid sphingomyelinase (50). Alternatively, macrophages could sequester protein VI into lumenal vesicles of endosomes, akin to murine macrophages sequestering incoming herpes virus particles (72), or mount an oxygen radical response that restricts viral entry (73). After escape from endosomes, AdV particles navigate to the nucleus along microtubules and dock at the NPC for vDNA uncoating from the capsid (74). Although vDNA uncoating is efficient in hiPSC-derived macrophages, a significant fraction of free vDNA is misdelivered into the cytoplasm, a feature also observed with other cell types (45, 48, 52, 60). Likely, similar impediments restrict AdV entry into other types of macrophages.

### Restriction of immediate-early E1A transcription

Expression of viral DNA is controlled by transcription factors binding to regulatory sequences and enhancers, followed by the recruitment of co-activators or co-repressors that modulate chromatin structure and RNA polymerase activity (75). AdVs depend on host factors for activation of their immediate-early gene E1A. In bronchial epithelial cells and HDF-TERT, E1A-e/p activation is mainly mediated by GA-binding protein (GABP) binding sites in the E1A enhancer region (27, 76–78). Although transcripts for both subunits of GABP transcription factor are detectable in hiPSC-derived macrophages (42) (see Dataset A1 at https://doi.org/10.5281/zenodo.18656599), their FPKM counts are low (~10–40), and may account, at least in part, for the poor activity of the E1A-e/p.

iPSC-derived macrophages strongly restrict E1A expression of both species B and C AdVs, in stark contrast to tissue culture cell lines that rapidly accumulate high E1A transcript counts per cell. In primary human bronchial epithelial cells and HDF-TERT, E2F transcription factors bind to the E1A-e/p and mediate IFN-induced repression by recruiting a repressor complex composed of E2F/DP-1, retinoblastoma family members pRb/p107/p130, and class I histone deacetylases (27, 28). In hiPSC-derived macrophages, however, an IFN response is unlikely the main reason for poor E1A expression, since the JAK1/2 inhibitor ruxolitinib did not significantly improve E1A expression. The observation that AdV infection of hiPSC-derived macrophages is restricted independently of IFN signaling is in line with a clinical case report of an interferonopathic child showing life-threatening pneumonitis upon influenza virus infection, but sufficient control of AdV (79).

### E1A transactivation of the E1A enhancer/promoter

Regardless of the nature of E1A-e/p suppression, the restriction could be bypassed when E1A was expressed from coinfecting AdV-C5-CMV-E1A or helper-dependent AdV-C5-CMV-E1, transactivating the suppressed native E1A-e/p and enabling viral progeny production, though at a low level. This result corroborates the strong positive feedback of E1A proteins on their own expression (64) and implies epigenetic silencing and activation of the E1A-e/p, possibly involving both cellular and viral gene control (19, 23). The E1A-mediated transactivation of the E1A-e/p reported here is somewhat reminiscent of how the immediate-early transcription factors BZLF1 (Z) and BRLF1 (R) of Epstein-Barr virus (EBV) activate each other’s promoters of the latent vDNA in gastric cancer cells and thereby reactivate EBV (80, 81). Akin to E1A (26, 30), these data imply an initial activation of the Z and R promoters through changes of the cell state.

E1A transactivation was mirrored in our RNA-seq data and single-cell RNA-FISH experiments. Coinfection with AdV-C5-CMV-E1A imposed an S-phase-like cell state and allowed progeny production at a low level, even after 2 days of the initial WT infection, thus raising the possibility that AdV-C5 might establish persistent infection in macrophages. Coinfection upregulated E2F1, E2F2, CCNE1, and PCNA transcripts, possibly orchestrated by E1A through neutralization of retinoblastoma protein-mediated repression of E2F-regulated genes required for S-phase transition (19, 82). In contrast, the cells infected by WT virus alone did not show any upregulation of E2F1, E2F2, or CCNE1 by 27 h pi, but displayed significant upregulation of the p21 cyclin-dependent kinase inhibitor 1 (CDKN1A). The progeny production from WT and AdV-C5-CMV-E1A coinfection was low, on average about eight infectious units per cell at 74 h pi. This suggests that hiPSC-derived macrophages likely impose additional restrictions beyond E1A on the nuclear phase of the infection, in line with studies in lymphoma-derived U-937 monocyte-like cells (83). The observation that the AdV-C5 E1A expression also transactivated the species B E1A-e/p suggests that there could be cross-species AdV interactions in persistently coinfected cells.

### Innate immune responses to dormant and progressing AdV infections

Macrophages are sentinels against invading viruses, including AdVs. For example, in alveolar murine macrophages, AdV-C5 triggers cGAS/STING and a proinflammatory cytokine response (34, 36, 45); in PMA-differentiated THP-1 cells and human monocyte-derived macrophages, AdV-C5 or B3 induces NLRP3 (NOD-, LRR-, and pyrin domain-containing protein 3) inflammasome (84); and in primary human macrophages, antibody-coated AdV-C5 triggers the NLRP3 inflammasome (85).

Our analyses of the host innate immune response to AdV in the hiPSC-derived macrophage model indicated that dormant single WT and progressing coinfections impose divergent cell states. The restricted single WT infections upregulated several chemokine transcripts and TNFSF10 (TRAIL) more strongly than the progressing WT and AdV-C5-CMV-E1A coinfections. The upregulated abundant chemokine transcripts included CXCL8, CXCL10, CXCL11, CCL3, CCL4, and CCL8, which have been described as common proinflammatory cytokines to AdV or its vectors (86, 87). Cytokine release assays confirmed CXCL8 secretion from the infected hiPSC-derived macrophages, albeit with clonal variability reflecting subtle heterogeneity in CXCL8 secretion. Although induction of chemokine transcripts in general was more robust in the restricted single WT infections, both single and coinfections shared strong upregulation of CXCL10 and CXCL11 transcripts, known to be potent chemoattractants of T cells (88). The RNA-seq data also indicated strong upregulation for IFN transcripts, especially in the coinfection setting, but the overall transcript counts were low, consistent with low levels of IFN in the culture supernatants possibly originating from just a few producer cells.

In the *in vivo* scenario of an acute AdV airway infection, tissue-resident macrophages or those invading the site of infection engulf virus particles (34), but they might restrict productive infection by silencing early viral gene expression. Their dormant AdV genomes would be prone to epigenetic reactivation, as shown in this study by ectopic E1A transactivation *in vitro*. Yet, other signaling pathways through proinflammatory cytokines (89) conceivably affect DNA methylation or histone modification in the E1A-e/p. In fact, cytokine receptor signaling induces contextually unique transcriptional and epigenetic environments (90). Likewise, superinfecting viruses, such as herpesviruses, alter the epigenetic landscape of the host (91), and, by inference, could reactivate dormant AdV genomes.

### Limitations

The study here offers a novel approach for mechanistic insights into epigenetic reprogramming of viral infections and gene delivery into immune-naive human macrophages. It is feasible that changes to the cell state akin to those imposed by coinfection reactivate dormant AdV genomes. hiPSC-derived macrophages are readily available from genetically different backgrounds and allow for both controlled repression and reactivation of AdV infection. While the repressed and activated infection states are amenable to epigenetic and chemical manipulation, differentiated macrophages are difficult to genetically edit due to their noticeable innate defense against viral and non-viral transduction. Although gene knockouts are possible in hiPSCs, they are limited to the genes that are not essential for macrophage differentiation. In addition, the pronounced phagocytic activity of single or coinfected hiPSC-derived macrophages limits their *ex vivo* lifespan to 4–5 days and requires adaptation for prolonged studies of viral persistence.

## MATERIALS AND METHODS

### iPSC lines and embryonic body factories

The hiPSC lines SFC854-03-02 (short 854-2), SFC840-03-03 (short 840-3), and SFC856-03-04 (short 856-4) are described in (92, 93) and can be obtained from ebisc.org (European Bank for Induced Pluripotent Stem Cells) with cell codes STBCi026-A (840-3), STBCi066-A (854-2), and STBCi063-A (856-4). The 854-2, 840-3, and 856-4 macrophage precursors were grown and differentiated into EB factories as previously described (41, 42). WTB6 and DW4 hiPSC clones are described in references 94, 95 and derived from EB factories obtained from the hiPSCore facility (Institute for Regenerative Medicine, University of Zurich). Medium from EB factories containing macrophage precursors was collected weekly, with concomitant replenishing of cultures with fresh medium. Harvested medium was passed through a 40 µm cell strainer (BD Falcon 352,340), cells were pelleted at 200 × *g* for 5 min, washed once with phosphate-buffered saline (PBS), plated in X-VIVO15 macrophage medium (43), and differentiated for 7 days, with one change of medium after 3 to 4 days. Since cells differentiated in X-VIVO15 macrophage medium have an elevated basal expression of select antiviral genes, an alternative OXM macrophage medium was tested as well (43). OXM medium was used for differentiation of the macrophages in the experiments shown in Fig. 4B, C, 5B and C and Fig. S1A and B with half of the medium replenished by fresh medium on day 3 or 4. A549 human lung epithelial carcinoma cells (American Type Culture Collection, ATCC), human embryonic retinoblast 911 cells (HER-911), HeLa (ATCC), and HEK-293 cells, as well as KB cells (a subline of HeLa cells), were maintained in DMEM (Sigma-Aldrich, D6429) supplemented with 7.5% fetal calf serum (FCS; Gibco/Thermo Fisher Scientific, 10270106) and 1% non-essential amino acids (Sigma-Aldrich, M7145).

### Viruses

WT-AdV-C5 was grown in A549 cells. EdC-labeled AdV-C5 was produced in A549 in the presence of 2.5 µM EdC (Jena Bioscience CLK-N003-10) added at 14 h pi (48). The non-replicating AdV-C5_dE1_GFP_dE3 vector (96, 97), which has E1/E3 deletions and expresses enhanced green fluorescent protein (GFP) from a CMV major immediate-early e/p, was grown in HER-911 cells. The penetration-deficient TS1-AdV-C2 (53) was grown in KB cells at the restrictive temperature of 39°C. In AdV-C5-CMV-E1A, the E1A/E1B transcription units are deleted and replaced by the E1A coding region under the control of CMV-e/p (27). AdV-C5-CMV-E1A was grown in HEK-293 cells. In AdV-C5-E1A-2A-GFP and AdV-B35-E1A-2A-GFP, E1A and GFP are under the native E1A-e/p and separated by the picornavirus-derived 2A sequence, which enables translation of both E1A and GFP proteins through ribosomal skipping (52, 67). Helper-dependent HD_CMV-E1 contains only E1A and E1B transcription units. All viruses were purified on CsCl gradients as previously described (98) and dialyzed against 10 mM Tris-HCl pH 8.1, 150 mM NaCl, and 1 mM MgCl_2_ for ~24 h with one change of buffer using Slide-A-Lyzer dialysis cassettes (10,000 MWCO, Thermo Fisher Scientific, 66383). Glycerol was added to a final concentration of 10%, and the viruses were stored in aliquots at −80°C. Absorbance measurements at 260 nm were used for determination of the number of v.p. per mL (99) and for normalizing input virus amounts in experiments presented in Fig. 1. Alternatively, the input virus amounts were normalized by determining median values for cell-associated v.p. after 60-min incubation on A549 cells at 37°C (Fig. 4, 6 and 7). Atto 565-labeled WT-AdV-C5 and Alexa Fluor 488-labeled TS1-AdV-C2 were prepared as previously described (98, 100) using Atto 565 NHS ester (Sigma 72,464) and Alexa Fluor 488 TFP ester (Thermo Fisher Scientific, A37570), respectively. Excess dye was removed with Zeba Spin Desalting 40K MWCO columns (Thermo Fisher Scientific, A57756).

### Cloning and production of helper-dependent AdV-C5-CMV-E1

For production of helper-dependent AdVs, the producer cell line 116 (kindly provided by Philip Ng, Baylor College of Medicine, Houston, USA) (101) was grown in MEM (Thermo Fisher Scientific), supplemented with 10% FCS, 2 mM glutamine (Sigma-Aldrich), and 100 µg/mL Hygromycin B (Thermo Fisher Scientific). Helper-dependent AdV encoding the desired payload was produced according to the iMATCH technology (102). First, the payload was subcloned into the shuttle plasmid pUniversal via Gibson Assembly (GA) as previously described (102) using the HiFi DNA Assembly Master Mix (New England Biolabs). To generate a pUniversal plasmid that drives E1A expression under the control of a CMV-e/p, the AdV-C5 E1 sequence was PCR amplified from nucleotide 560 to nucleotide 4344 (according to the reference AdV-C5 genome AC_000008) using the forward (TTTAGTGAACCGTCAGATCCTCGAGTATGAGACATATTATCTGCCACGGA) and reverse (AGATGGCTGGCAACTAGAAGGCACAGGATTGGCAATCAGCTTGCTACTGA) primers (template hybridization sequence is underscored) and purified WT-AdV-C5 genome as a template. The PCR product with suitable homology overlaps was subcloned via GA into a linear pUniversal plasmid that contained a CMV-e/p. Next, the pUniversal plasmid (103) was linearized to liberate suitable homologous sequence overlaps to subclone the transgene by GA into *AscI*-linearized HC-AdV backbone plasmid (pC4 hSU, 103). The pC4 hSU plasmid sequence encoding the E1 expression cassette was confirmed via Sanger sequencing. The resulting helper-dependent CMV-E1 virus was amplified and produced as previously described (102). Amplification was carried out in the producer cell line 116 using a human AdV-C5 helper vector (HV) without capsid modification. The amplified virus was harvested from 20 × 15 cm dishes, washed in ice-cold PBS, and extracted from the producer cells by three freeze-thaw cycles in liquid nitrogen and a 37°C water bath. The cleared cell lysate was loaded on a two-step CsCl density gradient (1.25 and 1.35 g/cm^3^), and upon ultracentrifugation for 2 h (280,000 × *g*, 12°C), AdV-C-CMV-E1 virions were separated from cellular debris and empty viral particles, and the virus band was transferred to a four-step CsCl density gradient (1.29 g/cm^3^ to 1.35 g/cm^3^) to further separate the AdV-C-CMV-E1 virions from HV particles and ultracentrifuged (280,000 × *g*, 12°C, 20–24 h). Banded AdV-C-CMV-E1 virions were collected, dialyzed against storage buffer (20 mM HEPES pH 8.1, 150 mM NaCl, 1 mM MgCl_2_) and snap-frozen in liquid nitrogen after the addition of glycerol to a final concentration of 10%.

### Virus binding assay

Macrophage precursor cells were seeded on a 96-well imaging plate at 40,000 cells/well and differentiated into macrophages as described above. Cells were pretreated with soluble AdV-C5 fiber knobs (104) at final concentration 1 µg/mL for 30 min on ice in X-VIVO15 macrophage medium supplemented with 0.2% bovine serum albumin (BSA). Subsequently, Atto 565-labeled AdV-C5 (MOI ~350,000; physical v.p.) was added to cells and incubated on ice for 60 min. Unbound viruses were removed, and cells were washed once with PBS and fixed with 3% PFA/PBS for 20 min at room temperature. Nuclei were stained with DAPI using PBS containing 0.1% Triton X-100. Alexa Fluor 647 NHS ester (A20006, Thermo Fisher Scientific; 0.5 µg/mL in PBS for 10 min) was used for staining of the cell area. Imaging was carried out with a Leica SP5 confocal laser scanning microscope using 63× magnification oil objective with numerical aperture (NA) 1.4 and zoom factor 2. Stacks were recorded at 1 µm intervals with sequential acquisition using between-stacks switching mode and 3× frame averaging for the virus signals. The Leica SP5 confocal microscope was maintained by the Center for Microscopy and Image Analysis, University of Zurich. Custom-programmed CellProfiler pipelines were used to score the number of cell-associated v.p. from maximum projections of image stacks. The resulting data were sorted using Knime Analytics Platform, and GraphPad Prism was used to create the scatterplots.

### Infection assays

Macrophage precursor cells were seeded on 96-well imaging plates (Greiner Bio-One, 655090) at 40,000 cells/well and differentiated into macrophages as described above. Cells were incubated with AdV-C5 at an MOI of ~10,000–50,000 (physical v.p./cell) for 6 to 15 h, unbound viruses were removed, and incubation continued in fresh medium for the indicated total times pi (exact MOI and incubation times are indicated in the figure legends). In Fig. 4B and Fig. S1A and B, the cells were pretreated with 2 µM ruxolitinib (InvivoGen, RUX-42-01; control cells treated with equal volume of the solvent DMSO) over two nights (Fig. 4B) or overnight (Fig. S1A and B). Ruxolitinib was present throughout the experiment. The effectiveness of the treatment was controlled by pre-incubating cells with 2 µM ruxolitinib or equal volume of the solvent DMSO for 180 min, followed by addition of 100 U of IFNα2a (pbl Assay Science, 11,100) for 26.5 h in the presence or absence of ruxolitinib. Induction of the ISG ZNFX1 was probed by Western blotting using recombinant rabbit monoclonal anti-ZNFX1 (Abcam ab179452, EPR12330, final concentration 0.16 µg/mL) and mouse anti-β-tubulin (Amersham N357, final concentration 0.15 µg/mL) as primary antibodies, and horseradish peroxidase-conjugated anti-rabbit or anti-mouse (Cell Signaling Technology, #7074 and #7076, respectively, 1/4,000 dilutions) as secondary antibodies. The A549 infection assays were done with an MOI of ~135–540 (physical v.p.) using A549 growth medium supplemented with penicillin/streptomycin (Sigma P0781, final concentration 100 units penicillin and 100 µg streptomycin per mL), and cells were fixed 24 h pi.

Immunofluorescence staining of fixed cells was carried out as previously described (44) using anti-E1A M58 antibody (Thermo Fisher Scientific MA5-13643, 500 ng/mL) and secondary goat anti-mouse Alexa Fluor 488- or donkey anti-mouse Alexa Fluor 594-conjugated antibodies (Thermo Fisher Scientific, A-11029 and A-21203, respectively, final concentration 2 µg/mL). Nuclei were stained with 4’,6-diamidino-2-phenylindole (DAPI; 1 µg/mL). The plates were imaged with Molecular Devices automated ImageX-press Micro XL wide-field imaging system using 10× Plan Fluor objective (NA 0.3) and a single focal plane for both E1A and DAPI channels corresponding to a middle section of nuclei. In the case of AdV-C5_dE1_GFP_dE3, AdV-C5-E1A-2A-GFP, and AdV-B35-E1A-2A-GFP infections, the GFP signal was used for scoring infected cells. Images were analyzed using a custom-programmed CellProfiler pipeline (CellProfiler version 2.2.0 or 4.2.1, http://cellprofiler.org) (105). Nuclei were segmented with the DAPI image and mean nuclear E1A or GFP intensities over the DAPI masks were determined. JMP (JMP Statistical Discovery version 13–15) was used to determine the threshold for an infected cell (99.5% cutoff value from the non-infected control cells), and infection index, i.e., the number of infected cells over the total number of cells analyzed, was calculated using Knime Analytics Platform (https://www.knime.com/knime-analytics-platform). GraphPad Prism 7 or 10 (GraphPad Software, La Jolla, CA, USA) was used to create the graphs. Representative images were processed in Fiji (106), applying the same parameters of brightness and contrast to all images in the series.

### Endocytosis assay

Macrophage precursor cells were seeded on Alcian blue-coated coverslips in a 24-well plate format at 400,000 cells/well in X-VIVO 15 macrophage medium and differentiated into macrophages as described above. Cells were incubated with Atto 565-labeled AdV-C5 (MOI ~86,000, physical v.p.) for 60 min on ice. Unbound virus was removed, medium was switched to warm RPMI-1640 medium (Sigma R7388) supplemented with 0.2% BSA and penicillin/streptomycin, and cells were transferred to a 37°C water bath for 0 min or 15 min. Cells were returned to +4°C, washed once with RPMI-BSA medium, and incubated in RPMI-BSA medium containing 10% pooled human serum off the clot (Innovative Research Inc., ISER 10 mL) for 30 min on ice. Tagging of cell surface particles with mouse anti-hexon 9C12 antibody was carried out as described (44), except that the antibody was diluted in RPMI-BSA medium containing 10% human serum. The 9C12 antibody, developed by Laurence Fayadat and Wiebe Olijve, was obtained from Developmental Studies Hybridoma Bank, developed under the auspices of the National Institute of Child Health and Human Development. Secondary antibody staining was with anti-mouse Alexa Fluor 488-conjugated antibodies (final concentration 4 µg/mL) for 30 min in PBS containing 1% fetal calf serum. Nuclei were stained with DAPI and cell area with Alexa Fluor 647 NHS ester. Imaging was carried out with a Leica SP8 upright confocal laser scanning microscope using a 63× magnification oil objective (NA 1.4) and a zoom factor of 1.5. Stacks were recorded at 1 µm intervals with sequential acquisition using between-frames switching mode and 2× line accumulation and 3× frame averaging for the 9C12 and virus signals, respectively. Maximum projections of confocal stacks were analyzed by a custom-programmed MATLAB routine to score the number of cell surface-associated viruses (viruses with both Atto 565 and Alexa Fluor 488 signals) and internalized viruses (viruses with only Atto 565 signal) per cell (only cells having ≥5 v.p. were included in the analyses). The threshold for a 9C12-positive particle was determined by overlaying virus images with 9C12-stained images of non-infected cells, and the resulting 95% cutoff value of virus-associated 9C12 signal was used as a threshold. GraphPad Prism was used for making the scatterplots.

### Protein VI exposure

The experiment was carried out with Atto 565-labeled AdV-C5 as described above for the endocytosis assay, except that the internalization in the 37°C water bath was for 0 min, 15 min, or 30 min. Afterward, cells were fixed and immunostained with affinity-purified rabbit anti-VI antibodies (47) and secondary Alexa Fluor 488-conjugated goat anti-rabbit antibodies (Thermo Fisher Scientific, A11034; final concentration 4 µg/mL). Nuclei were stained with DAPI and cell area with Alexa Fluor 647 NHS ester. Samples were imaged with a Leica SP8 upright confocal laser scanning microscope as described above for the endocytosis assay. Maximum projections of confocal stacks were analyzed by a custom-programmed MATLAB routine to score the number of VI-positive v.p./cell (only cells with ≥6 v.p. were included in the analyses). The threshold value for a VI-positive particle was determined by placing a virus image on an antibody image obtained from non-infected cells and taking the 99% cutoff value of “virus-associated” VI signal as the threshold. GraphPad Prism was used for making the scatterplots. Representative images shown are maximum projections of the confocal stacks. The images were processed with Fiji.

### Streptolysin O (SLO) assay to detect virus particles penetrated into the cytoplasm

Macrophage precursor cells were seeded on Alcian blue-coated coverslips in a 24-well plate format at 280,000 cells/well in X-VIVO 15 macrophage medium and differentiated into macrophages as described above. Cells were incubated with Atto 565-labeled AdV-C5 (MOI ~12,900, physical v.p.) or Alexa Fluor 488-labeled TS1-AdV-C2 for 60 min at 37°C in X-VIVO 15 macrophage medium. Unbound viruses were removed, and incubation continued at 37°C in X-VIVO macrophage medium for 50 min; medium was replaced by plain DMEM containing 150 µg/mL cholesterol-methyl-β-cyclodextrin (Sigma C4951), and cells were incubated at 37°C for 15 min. Cells were then placed on ice, and SLO permeabilization was carried out as previously described (44) using pre-titrated amounts of SLO that enabled efficient cell permeabilization (SLO was from Sigma S5265-25KU, resuspended in 190 µL PBS containing 0.1% BSA and 1 mM DTT and stored in aliquots at −20°C). Permeabilized cells were incubated on ice for 60 min using mouse anti-hexon 9C12 (AdV-C5) and rabbit anti-Giantin (Abcam ab80864, final concentration 0.8 µg/mL) antibodies or rabbit anti-Alexa Fluor 488 for TS1-AdV-C2 (Thermo Fisher Scientific, A-11094, final concentration 0.8 µg/mL) and mouse anti-Giantin (final concentration 67 µg/mL; antibody kindly provided by Hans-Peter Hauri, Biocenter of the University of Basel) in SLO internalization buffer containing 5% pooled human serum off the clot. Cells were washed twice with excess SLO internalization buffer, fixed, and stained with secondary anti-mouse Alexa Fluor 488- and anti-rabbit Alexa Fluor 680 (Thermo Fisher Scientific, A-21076)-conjugated antibodies (final concentration 4 µg/mL; AdV-C5 sample) or with donkey anti-rabbit Alexa Fluor 594 (Thermo Fisher Scientific, A-21207) and goat anti-mouse Alexa Fluor 680 (Thermo Fisher Scientific, A-21057) antibodies (final concentration 4 µg/mL, TS1 sample), as described in reference 44. Nuclei were stained with DAPI. Control samples were fixed and permeabilized with Triton X-100 prior to antibody staining. Samples were imaged with a Leica SP5 confocal laser scanning microscope as described above for the virus binding assay, except that sequential acquisition was performed with between-frames switching mode and 3× frame averaging for virus or 9C12 or anti-Alexa Fluor 488 antibody signals (no frame averaging was needed for anti-Giantin signals). A custom-programmed CellProfiler pipeline was used to score the fraction of antibody-positive v.p., i.e., cytoplasmic v.p./cell. The threshold for an antibody-positive AdV-C5 particle was determined by placing a virus image on an antibody image obtained from non-infected cells and taking the 99.5% cutoff value of “virus-associated” antibody signal as the threshold. In the case of TS1, the virus-associated antibody signals from the SLO- and Triton X-100-permeabilized cells were combined, after which two peaks were visible in a histogram. The threshold value for an antibody-positive virus particle was chosen from the area between the two peaks after manually certifying from images that the chosen threshold clearly distinguished antibody-positive and -negative v.p. Only properly permeabilized cells, i.e., Giantin-positive cells, and cells having ≥5 v.p. were included in the analyses. GraphPad Prism was used for making the scatterplots, and representative images shown were processed with Fiji.

### Uncoating of incoming vDNA

Macrophage precursor cells were seeded on Alcian blue-coated coverslips in a 24-well plate format at 200,000 cells/well in X-VIVO 15 macrophage medium, and differentiated into macrophages as described above. Cells were incubated with EdC-labeled AdV-C5 (MOI ~12,400, physical v.p.) at 37°C for 60 min in X-VIVO 15 macrophage medium. Unbound viruses were removed, and incubation was continued for an additional 10 min or 360 min in X-VIVO15 macrophage medium. Cells were fixed and immunostained with the anti-hexon 9C12 antibody and secondary Alexa Fluor 488 (70-min sample)- or Alexa Fluor 594 (420-min sample)-conjugated secondary antibodies. The vDNA in the 420-min sample was tagged with azide-Alexa Fluor 488 (Thermo Fisher Scientific, A10266) using a click reaction (48). Nuclei were stained with DAPI. Samples were imaged within 24 h with a Leica SP5 confocal laser scanning microscope using a 63 × magnification oil objective (NA 1.4) and a zoom factor of 2.5. Stacks were recorded at 1 µm intervals with sequential acquisition using between-frames switching mode and 3× frame averaging for the virus capsid and vDNA signals. The use of the sensitive Leica HyD hybrid detector in the normal mode was essential for the detection of the vDNA signals. Maximum projections of confocal stacks and a custom-programmed CellProfiler pipeline were used for scoring cell- and nucleus-associated v.p. and for determining vDNA-associated capsid signals. For vDNA foci segmentation, the vDNA images were first processed with the Fiji plugin Rolling Ball Background Subtraction, with a rolling ball radius of five pixels. Proper nucleus and cell segmentation was controlled and manually adjusted if necessary. The threshold value for a hexon-positive vDNA was determined manually by comparing values of vDNA hexon signals that were visually judged from the images to be either positive or negative. The resulting data were sorted using the Knime Analytics Platform, and GraphPad Prism was used to create the scatterplots. Representative images shown were processed with Fiji.

### Virus progeny production from infected macrophages

Infection progression to a late phase and virus progeny production were tested in 856-4 and 840-3 cells. Macrophage precursor cells of clone 856-4 were seeded on 96-well imaging plates at 50,000 cells/well in X-VIVO15 macrophage medium and differentiated into macrophages as described above. The cells were incubated with AdV-C5, AdV-C5-CMV-E1A, or both viruses (MOI ~42,000 physical v.p./cell in the single infections and ~21,000 for each virus in the coinfection) in the X-VIVO15 macrophage medium for 8.5 h; unbound viruses were removed; cells were washed with excess medium; and incubation was continued in fresh X-VIVO15 macrophage medium for a further 42.5 h (late phase) or 65.5 h (progeny production; Fig. 5A and B). Progression of the infected cells to late infection phase was scored using antibodies against the late protein VI and secondary anti-rabbit Alexa Fluor 488-conjugated antibodies, as well as DAPI for the cell nucleus. Samples were analyzed as described above for the infection assays. For testing virus progeny production, cell- and medium-associated particles were collected at 74 h pi. Medium was centrifuged at 400 × *g* for 5 min. Cells were scraped into 10 mM Tris-HCl (pH 8.1 at room temperature) and freeze-thawed three times using liquid nitrogen for freezing and thawing at 37°C in a water bath. The cell lysates were extracted with an equal volume of Freon 113 (1,1,2-trichloro-1,2,2-trifluorethane), subjected to centrifugation at 4,000 × *g* for 5 min at +4°C, and the aqueous phase was collected, i.e., cell-associated material. Equal volumes of clarified culture supernatant and cell-associated material were titrated on A549 cells, and infectious units were scored by anti-VI immunostaining at 32 h pi as described above for the infection assays.

To test whether AdV-C5-CMV-E1A superinfection could activate a dormant WT-AdV-C5, 840-3 macrophage precursors (250,000 cells/well) were seeded on a 24-well plate in X-VIVO15 macrophage medium and differentiated into macrophages. The cells were incubated with AdV-C5 (MOI ~42,000 physical v.p./cell) in X-VIVO15 macrophage medium for 9 h; unbound viruses were removed; cells were washed with excess medium; and incubation was continued in fresh X-VIVO15 macrophage medium for a further 39.5 h. Superinfection was carried out with AdV-C5-CMV-E1A (MOI ~42,000 physical v.p./cell) in X-VIVO15 macrophage medium for 7 h. After removal of unbound viruses and washing with excess medium, incubation was continued in fresh X-VIVO15 macrophage medium for a further 68 h. Cell- and medium-associated progeny viruses were collected and titrated on HeLa cells as described above.

### RNA-seq

RNA sequencing (RNA-seq) was performed by the Functional Genomics Center Zurich (FGCZ) at the University of Zurich and ETH Zurich. One million clone 856-4 cells per well were seeded on a 6-well plate in X-VIVO15 macrophage medium, and cells were differentiated into macrophages as described above. Virus infections were carried out in the same medium. Macrophages were incubated with either WT-AdV-C5 alone or with WT plus AdV-C5-CMV-E1A for 12 h, unbound viruses removed, and cells were incubated in fresh medium for an additional 0 or 15 h. To ensure similar MOI for the infections (total input v.p./cell), the input virus amounts were normalized by determining median values for cell-associated v.p. after 60 min of incubation on A549 cells at 37°C. The MOI used was ~42,000 physical v.p./cell. Non-infected cells served as controls for both time points. All samples were done in triplicate. Cells were briefly rinsed with PBS-0.5 mM EDTA and incubated in PBS-EDTA for 2–4 min at 37°C to detach the cells, centrifuged at 215 × *g* for 7 min, and the cell pellet resuspended in 300 µL Trizol and incubated at room temperature for 15 min, followed by centrifugation at 16,000 × *g* for 30 s. Supernatant was used for RNA extraction with the Zymo Research Quick-RNA Miniprep Plus Kit (Zymo Research R1058) according to the manufacturer’s protocol. Extracted RNA was prepared for sequencing using the Illumina TruSeq Stranded Total RNA Kit (ribosomal depletion with Ribo-Zero rRNA Removal Kit) following the manufacturer’s protocol. Sequencing was performed on the Illumina NovaSeq 6000 using the S1 Reagent Kit v1.5 (100 cycles), as per manufacturer’s protocol. Demultiplexing was performed using the Illumina bcl2fastq software (v1.10). Per-sample read numbers ranged from 27.4 million to 54 million reads. RNA sequencing analysis was performed using the SUSHI framework (107). Read quality was inspected using FastQC, and sequencing adaptors were removed using fastp (108). RNA-seq reads were mapped simultaneously to the GENCODE human genome build GRCh38 patch 13 (annotation release 37-2021-05-04) (109) and the adenovirus 5 (AC_000008.1) whole genome using the STAR aligner (110). Counting of gene-level expression values was done using the “featureCounts” function of the R package Rsubread (111). Differential expression analyses were performed with the edgeR Bioconductor package (112). All R functions were executed on R version 4.0.4 (R Core Team, 2020) and Bioconductor version 3.12. Visualization of the RNA-seq results was performed with Explore DE and MultiDEG interactive Shiny Apps provided by the FGCZ (Peter Leary and Hubert Rehrauer, Zenodo.org, https://doi.org/10.5281/zenodo.13927692).

### RT-qPCR

RNA of the 856-4 infections was the same as used in the RNA-seq. For 854-2 infections, 250,000 cells per well were seeded on a 24-well plate in X-VIVO15 macrophage medium, and cells were differentiated into macrophages as described above. Virus infections were carried out in the same medium. Macrophages were incubated with either WT-AdV-C5 alone or with WT plus AdV-C5-CMV-E1A for 9 h (total MOI in both infections ~8,400 v.p./cell), unbound viruses were removed, and cells were incubated in fresh medium for an additional 15 h or 36.5 h. Cell culture medium from 856-4 and 854-2 infections was collected, clarified by centrifugation at 215 × *g*, HEPES-KOH was added to a final concentration of 10 mM, and samples were flash-frozen in liquid nitrogen and stored at −80°C until analysis (see “Cytokine secretion,” below). Total RNA from cells was prepared as described above for the RNA-seq. cDNA was synthesized in a 20 µL reaction mix containing 100 ng RNA, 0.5 mM dNTPs (Thermo Fisher Scientific, R0193), 10 mM dithiothreitol (DTT), 25 µg/mL oligo(dT)_15_ primer (Promega, C1101), 1× M-MLV reverse transcriptase (RT) buffer (Promega M531A), 100 units M-MLV RT (Promega, M1701; no RT control had water instead), and nuclease-free water (Thermo Fisher Scientific, 10977035) to fill the volume to 20 µL. Reaction mixtures without DTT and RT were first incubated at 70°C for 10 min, followed by 2.5 min at 62°C and 1 min at 42°C in an Eppendorf Nexus GX2 Mastercycler (Sigma, EP6336000040). At this point, the thermocycler was paused, and DTT and RT were added, and incubation at 42°C was then continued for 59 min. Nuclease-free water (30 µL) was added to the samples, and after 1 min at 42°C, temperature was switched to 95°C for 10 min, followed by cooling of samples to +4°C. The cDNA was amplified by PCR in a 10 µL reaction mix containing 5 µL Applied Biosystems PowerTrack SYBRGreen Master Mix (Thermo Fisher Scientific, A46109), 3.5 µL nuclease-free water, 0.5 µL 10 µM primer mix (primers listed in Table S1; https://doi.org/10.5281/zenodo.18338932), and 1 µL cDNA. Thermal cycling was performed with QuantStudio 3 Real-Time PCR System thermocycler (Thermo Fisher Scientific) in Applied Biosystems MicroAmp Optical 96-well reaction plates (Thermo Fisher Scientific, N8010560) using the following cycle conditions: 95°C for 10 min, followed by 40 cycles at 95°C for 30 s and at 60°C for 1 min. Results were analyzed using QuantStudio Design and Analysis Software v1.5.1. Relative mRNA levels compared to control cells were calculated using the 2^ΔΔCt^ method (113), using GAPDH as an internal control.

### Cytokine secretion

Frozen clarified 856-4 and 854-2 culture supernatants from the RT-qPCR experiments were thawed, and virus was inactivated by a 13-hour incubation in 0.5% β-propiolactone at + 4°C, followed by a 2-hour incubation at 37°C. Cytokines secreted into the culture medium were assayed by Bio-PLEX Pro Human Inflammation Panel 1, 37-plex assay kit (Bio-Rad 171AL001M) according to the manufacturer’s instructions, using undiluted culture supernatants and a Luminex 200 for plate reading.

### RNA FISH with branched DNA signal amplification

Macrophage precursor cells were seeded on 96-well imaging plates at 40,000–60,000 cells per well in X-VIVO15 or OXM macrophage medium and differentiated into macrophages as described above. Virus infections were carried out in the same medium (MOI and incubation times are indicated in the figure legends). The RNA FISH was carried out using the ViewRNA HC screening assay system (Thermo Fisher Scientific) and a custom-made probe against AdV-C5 E1A mRNAs (VF1-15472-01, type 1 probe, Alexa Fluor 546), as previously described (26). Nuclei were stained with DAPI, and cell area with Alexa Fluor 680 NHS Ester (Thermo Fisher Scientific, A37574). Samples were imaged with an ImageXpress Micro confocal imaging system using a 40× Plan Apo Lambda objective (NA 0.95), and confocal stacks were acquired for all channels. Transcript foci were scored per cell from maximum-projection images using custom-programmed CellProfiler pipelines. To improve the transcript foci segmentation, images were first processed with the Fiji plugin Rolling Ball Background Subtraction, using a rolling ball radius of five pixels. The transcript foci within the same cell had different intensities, and a relatively stringent thresholding for foci detection was used, which excluded some of the dimmest foci. Furthermore, segmentation of individual foci in a cluster of foci was difficult, and thus the shown transcript counts per cell are, to a certain degree, underestimations, but are representative of relative differences between samples. GraphPad Prism was used to create the scatterplots.

## Data Availability

The RNA-seq data are deposited in NCBI’s Gene Expression Omnibus (114) and are accessible through GEO Series accession number GSE317750. Raw data for the figures, as well as Table S1 and Datasets A2 and A3, are available on Zenodo (10.5281/zenodo.18338933). Dataset A1 (differentially expressed genes in AdV-C5-infected hiPSC-derived macrophages in comparison to non-infected control cells) is available on Zenodo (10.5281/zenodo.18656599).

## SUPPLEMENTAL MATERIAL

Supplemental material jvi.00392-26-s0001.pdf Fig. S1 to S3; captions for Datasets A1 to A3.

ASM does not own the copyrights to Supplemental Material that may be linked to, or accessed through, an article. The authors have granted ASM a non-exclusive, world-wide license to publish the Supplemental Material files. Please contact the corresponding author directly for reuse.
