# Supplementary material for: Transcriptional transactivation turns human iPSC-derived macrophages into an adenovirus-producing cell state
Source: J Virol. 2026 May 4;100(6):e00392-26. doi: 10.1128/jvi.00392-26 (PMC13288996; doi:10.1128/jvi.00392-26)
Supplement: Supplemental material — Fig. S1 to S3; captions for Datasets A1 to A3. [file jvi.00392-26-s0001.pdf]

Supplementary Figures and Captures to Supporting Datasets

Transcriptional transactivation turns human hiPSC-macrophages into an adenovirus producing cell state

Maarit Suomalainen, Walther Haenseler, Jonas Kolibius, Andreas Plückthun, Patrick Hearing and Urs F. Greber*

*Corresponding author: urs.greber@mls.uzh.ch; ORCID: 0000-0003-2278-120X

This PDF file includes:

Figures S1, S2, S3

Captures to Datasets A1 to A3

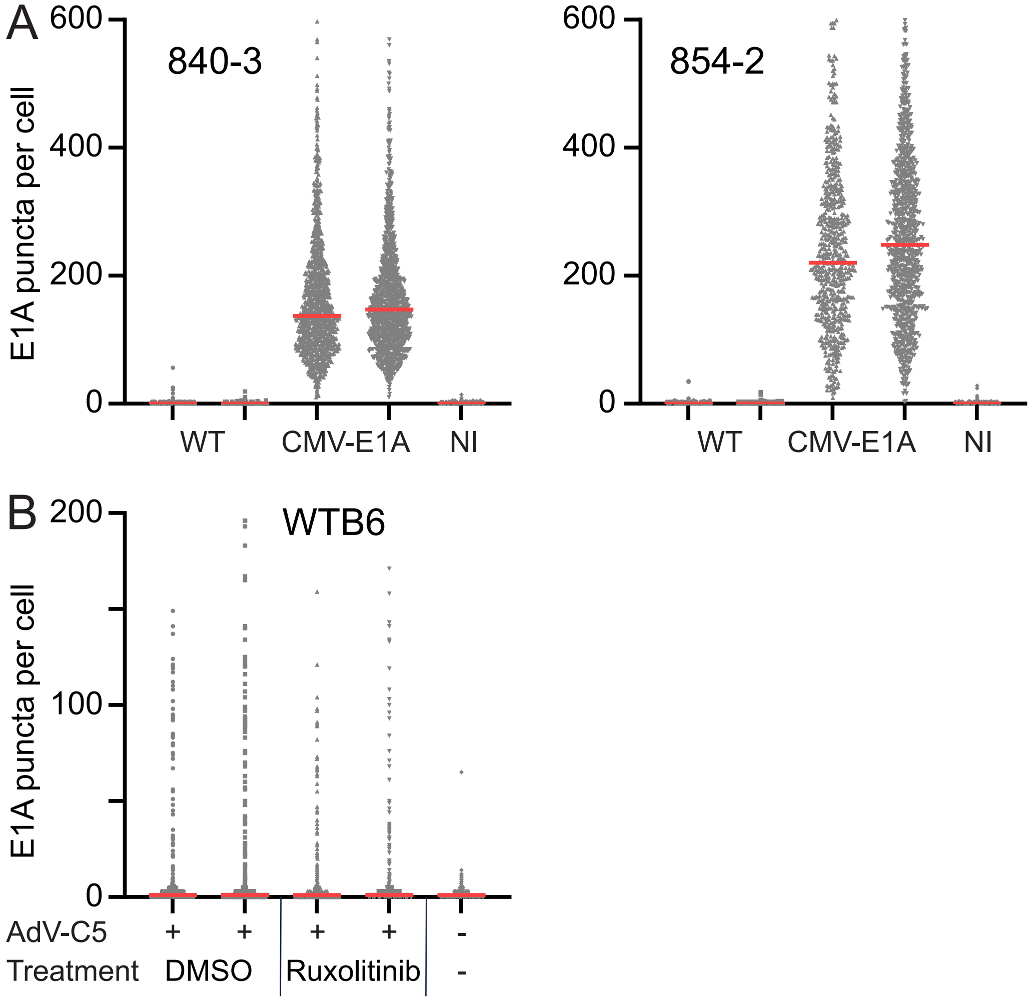

Suppl. Fig. 1. Replacement of native E1A-e/p by a CMV-e/p dramatically boosts the number of E1A transcripts in hiPSC-macrophages (related to Fig. 4)

A) Replacement of native E1A-enhancer/promoter (e/p) by a CMV-e/p dramatically boosts the E1A expression in hiPSC-macrophages. 840-3 and 854-2 cells were incubated with similar input virus amounts of AdV-C5 (WT) and AdV-C5-CMV-E1A (CMV-E1A) at high MOI ~87,500 v.p./cell for 15 h. Unbound virus was then removed and incubation was continued for additional 4 h. Noninfected cells (NI) were used as a control. E1A transcript counts per cell were analyzed by a single-cell, single-molecule RNA-FISH. One dot in the graph indicates transcript counts per a single cell, the two technical replicates of infected samples are shown separately. Horizontal bars represent median values. Over 600 cells were analyzed for each sample. Transcript counts > 100 are estimates due to difficulties of segmenting single transcript foci in

high expression cells. The AdV-C5-CMV-E1A infections are missing 19 (840-3) or 14 (854-2) data points because these were outside the upper limit of the y-axis. B) Poor activity of the native E1A e/p in hiPSC-macrophages is unlikely due to IFN-mediated repression. WTB6 cells were pretreated with the JAK1/2 inhibitor ruxolitinib (2 μM) or the DMSO solvent control over one night (23 h) prior to and during inoculation of WT at MOI ~14,000 v.p./cell for 7 h. Cells were analyzed at 27 h pi as in A). Y-axis limit was set to 200, which excluded total of 4 data points from the infected samples.

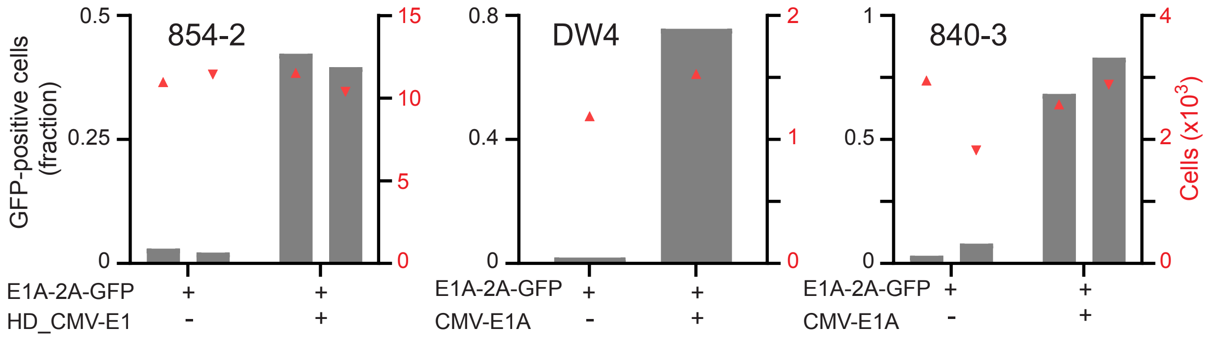

Suppl. Fig. 2: CMV-e/p-driven expression of E1A transactivates the native E1A e/p (related to Fig. 5)

Macrophages were inoculated with AdV-C5-E1A-2A-GFP (E1A-2A-GFP, native E1A e/p), or with E1A-2A-GFP plus HD_CMV-E1 or AdV-C5-in340-Δ2-CMV-E1A (CMV-E1A) at total MOI of ~ 38,400 (854-2), 51,000 (DW4) or 110,000 (840-3 coinfection; 840-3 single E1A-2A-GFP infection ~ 67,500) v.p./cell for 6.5 -9 h. The activity of the native E1A e/p was scored by analyzing the fraction of GFP-positive cells by wide field microscopy 31 h pi.

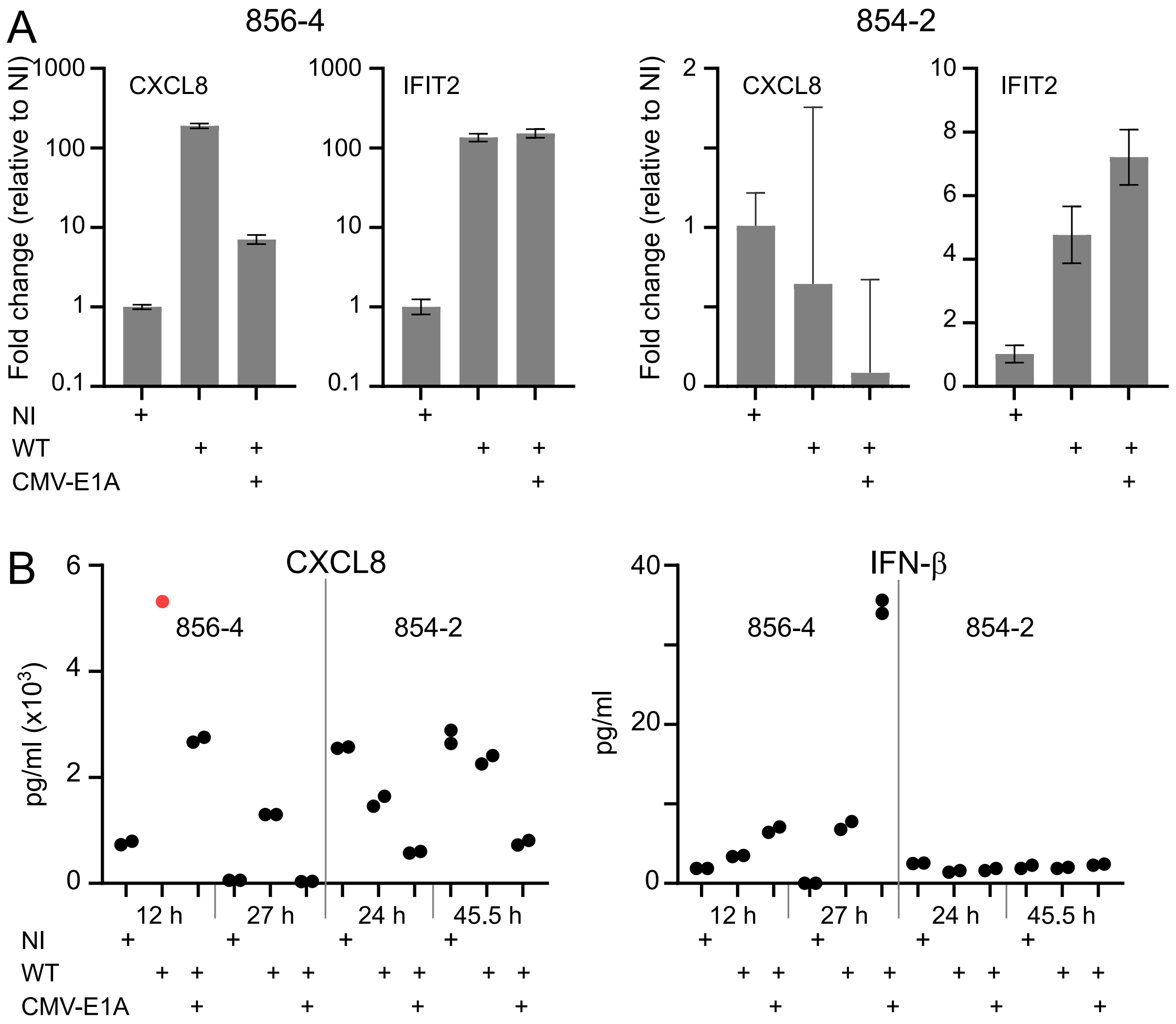

Suppl. Fig. 3: Host responses to AdV-C5 infection differ between 856-4 and 854-2 macrophages (related to Fig. 6)

A) RT-qPCR for CXCL8 and IFIT2 transcripts. Clone 856-4 macrophages were incubated with viruses for 12 h (total moi ~ 42,000 v.p./cell) and cell extracts were prepared at 27 h pi, whereas 854-2 cells were inoculated at lower moi (~8400 v.p./cell) for 9 h and analyzed at 24 h pi. GAPDH gene was used as an internal standard. Prolonging the 854-2 infection to 45.5 h pi did not yield higher induction for either CXCL8 or IFIT2 (data not shown). Note that the MOI for the 854-2 infection was five-fold lower than in the 856-4 infection, potentially explaining the lower fold induction of CXCL8 and IFIT2 in the 854-2 cells. **B)** CXCL8 and IFN-β secretion into the culture medium from the experiment described in A. The red dot indicates an approximate amount of secreted cytokine, as the value of the measurement fell above the standard curve.

Dataset A1: Summary of the DEGs based on RNAseq

The list contains the full data set from the RNAseq analyses from iPSC-derived 856-4 macrophages for WT single infections, WT/AdV-C5-CMV-E1A co-infections and noninfected cells at 12 and 27 h pi.

10.5281/zenodo.18656600

Dataset A2: Over-representation analyses (ORA) of top up- and downregulated biological process (BP) GO terms based on p-value

The file contains a list of genes including the GO annotation that are overrepresented in WT and WT/CMV coinfected iPSC-derived 856-4 macrophages at 12 and 27 h pi.

10.5281/zenodo.18338933

Dataset A3: Cytokine secretion from infected 856-4 and 854-2 macrophages using Bioplex assay

The file contains results from the full panel of Bio-PLEX Pro Human Inflammation Panel 1 37-plex assay.

10.5281/zenodo.18338933
